# The significance of cephalopod beaks as a research tool: An update

**DOI:** 10.3389/fphys.2022.1038064

**Published:** 2022-11-16

**Authors:** José C. Xavier, Alexey V. Golikov, José P. Queirós, Catalina Perales-Raya, Rigoberto Rosas-Luis, José Abreu, Giambattista Bello, Paco Bustamante, Juan C. Capaz, Valerie H. Dimkovikj, Angel F. González, Hugo Guímaro, Airam Guerra-Marrero, José N. Gomes-Pereira, Jorge Hernández-Urcera, Tsunemi Kubodera, Vladimir Laptikhovsky, Evgenia Lefkaditou, Fedor Lishchenko, Amanda Luna, Bilin Liu, Graham J. Pierce, Vasco Pissarra, Elodie Reveillac, Evgeny V. Romanov, Rui Rosa, Marjorie Roscian, Lisa Rose-Mann, Isabelle Rouget, Pilar Sánchez, Antoni Sánchez-Márquez, Sónia Seixas, Louise Souquet, Jaquelino Varela, Erica A. G. Vidal, Yves Cherel

**Affiliations:** ^1^ Department of Life Sciences, Marine and Environmental Sciences Centre/ ARNET–Aquatic Research Network, University of Coimbra, Coimbra, Portugal; ^2^ British Antarctic Survey, Natural Environment Research Council, Cambridge, United Kingdom; ^3^ GEOMAR Helmholtz Centre for Ocean Research Kiel, Kiel, Germany; ^4^ Instituto Español de Oceanografía (IEO,CSIC), Santa Cruz de Tenerife, Spain; ^5^ CONACYT -Tecnológico Nacional de México/I. T., Chetumal, Quintana Roo, Mexico; ^6^ Mola di Bari, Italy; ^7^ Littoral Environnement et Sociétés (LIENSs), UMR 7266 CNRS-La Rochelle Université, La Rochelle, France; ^8^ Institut Universitaire de France (IUF), Paris, France; ^9^ Center of Marine Sciences, University of Algarve, Campus de Gambelas, Faro, Portugal; ^10^ Department of Marine Science, Coastal Carolina University, Conway, SC, United States; ^11^ Instituto de Investigaciones Marinas (CSIC), Vigo, Spain; ^12^ IU-ECOAQUA, University of Las Palmas de Gran Canaria, Edf. Ciencias Básicas, Campus de Tafira, Las Palmas de Gran Canaria, Spain; ^13^ Atlantic Naturalist Association, Horta, Portugal; ^14^ National Museum of Nature and Science, Tokyo, Japan; ^15^ Centre for Environment, Fisheries and Aquaculture Science (CEFAS), Lowestoft, United Kingdom; ^16^ HCMR, Hellenic Centre for Marine Research, Athens, Greece; ^17^ Laboratory for Ecology and Morphology of Marine Invertebrates, A.N. Severtsov Institute of Ecology and Evolution of the Russian Academy of Sciences, Moscow, Russia; ^18^ Department of Ecology and Animal Biology, Faculty of Marine Sciences, University of Vigo, Vigo, Spain; ^19^ College of Marine Sciences, Shanghai Ocean University, Shanghai, China; ^20^ MARE—Marine and Environmental Sciences Centre/ARNET–Aquatic Research Network, Laboratório Marítimo da Guia, Faculdade de Ciências, Universidade de Lisboa, Cascais, Portugal; ^21^ Centre Technique de Recherche et de Valorisation des Milieux Aquatiques (CITEB), Le Port, Île de la Réunion, France; ^22^ Centre de Recherche en Paléontologie-Paris (CR2P), CNRS, Sorbonne Université, Paris, France; ^23^ University of South Florida, College of Marine Science, St. Petersburg, FL, United States; ^24^ Institut de Ciènces del Mar, CSIC, Psg. Marítim de la Barceloneta, Barcelona, Spain; ^25^ Universidade Aberta, Rua Escola Politécnica, Lisboa, Portugal; ^26^ Department of Mechanical Engineering, Faculty of Engineering Science, University College London, London, United Kingdom; ^27^ Center for Marine Studies—Federal University of Parana (UFPR), Pontal do Paraná, PR, Brazil; ^28^ Centre d’Etudes Biologiques de Chizé, UMR 7372 du CNRS-La Rochelle Université, Villiers-en-Bois, France

**Keywords:** cephalopod ecology, beak taxonomy/composition/morphology/microstructure/paleontology, cephalopod trophic dynamics, cephalopod population dynamics, cephalopod ecotoxicology

## Abstract

The use of cephalopod beaks in ecological and population dynamics studies has allowed major advances of our knowledge on the role of cephalopods in marine ecosystems in the last 60 years. Since the 1960’s, with the pioneering research by Malcolm Clarke and colleagues, cephalopod beaks (also named jaws or mandibles) have been described to species level and their measurements have been shown to be related to cephalopod body size and mass, which permitted important information to be obtained on numerous biological and ecological aspects of cephalopods in marine ecosystems. In the last decade, a range of new techniques has been applied to cephalopod beaks, permitting new kinds of insight into cephalopod biology and ecology. The workshop on cephalopod beaks of the Cephalopod International Advisory Council Conference (Sesimbra, Portugal) in 2022 aimed to review the most recent scientific developments in this field and to identify future challenges, particularly in relation to taxonomy, age, growth, chemical composition (i.e., DNA, proteomics, stable isotopes, trace elements) and physical (i.e., structural) analyses. In terms of taxonomy, new techniques (e.g., 3D geometric morphometrics) for identifying cephalopods from their beaks are being developed with promising results, although the need for experts and reference collections of cephalopod beaks will continue. The use of beak microstructure for age and growth studies has been validated. Stable isotope analyses on beaks have proven to be an excellent technique to get valuable information on the ecology of cephalopods (namely habitat and trophic position). Trace element analyses is also possible using beaks, where concentrations are significantly lower than in other tissues (e.g., muscle, digestive gland, gills). Extracting DNA from beaks was only possible in one study so far. Protein analyses can also be made using cephalopod beaks. Future challenges in research using cephalopod beaks are also discussed.

## Introduction

**FIGURE 1:**
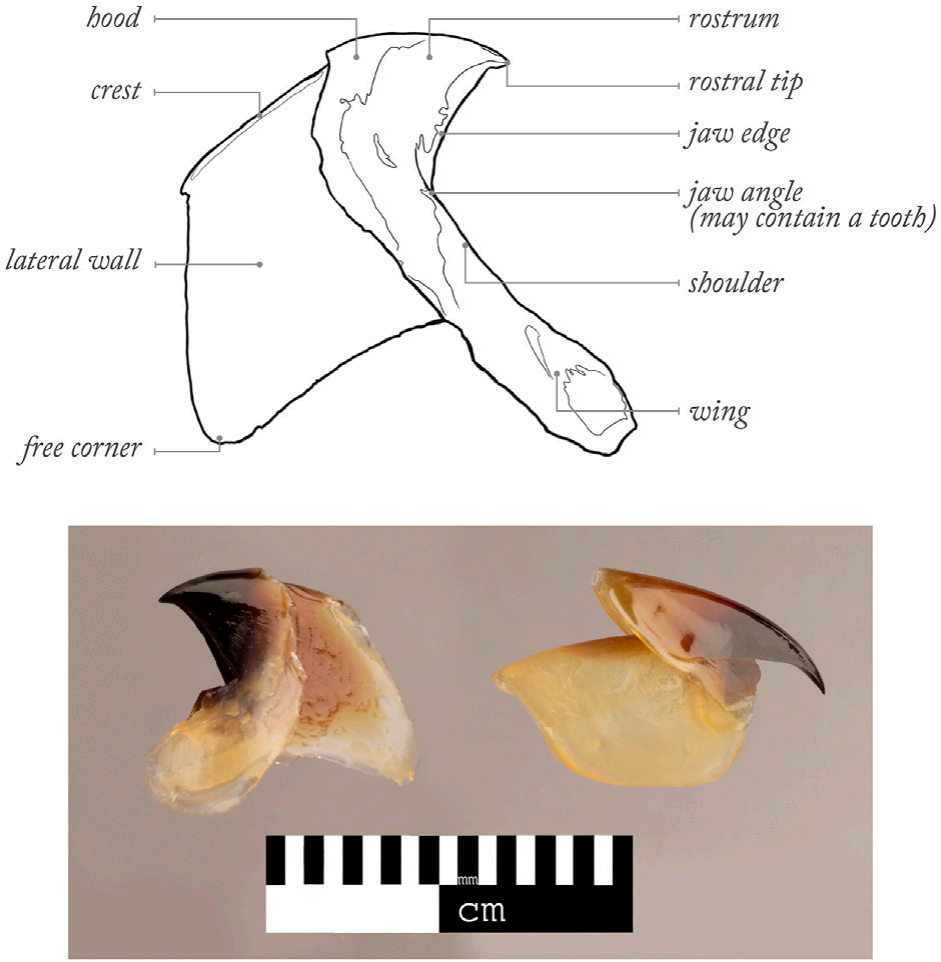
Examples of profiles of lower (on the left) and upper (on the right) beaks of cephalopods and the principal terms used to characterize decapod beaks (Xavier and Cherel (2021) following Clarke (1986)).

**FIGURE A1:**
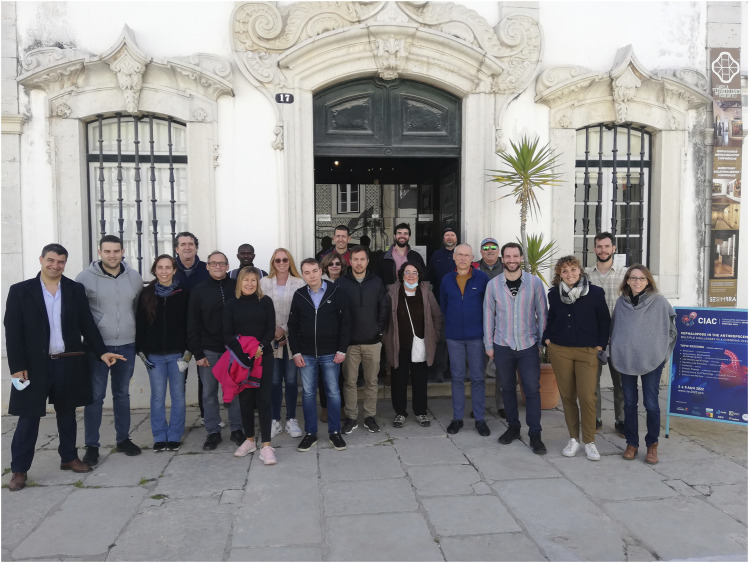
Group photo of the participants, in person, of the CIAC 2022 beak workshop (Sesimbra, Portugal).

**FIGURE A2:**
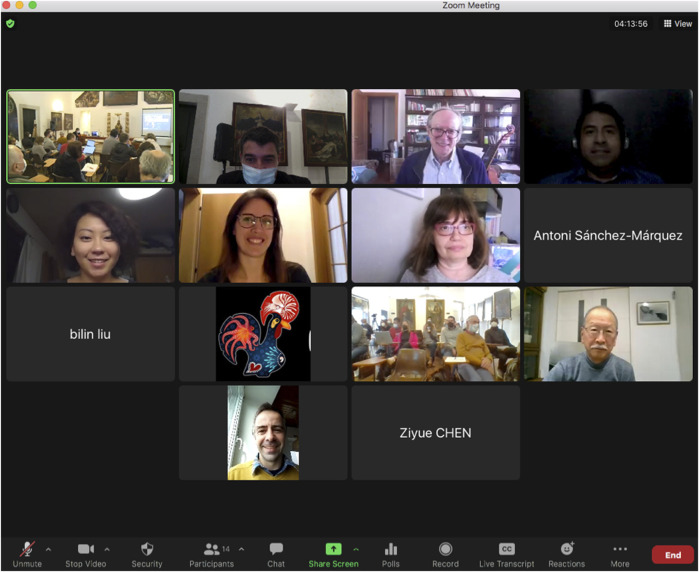
Group photo of the participants online of the CIAC 2022 beak workshop (Sesimbra, Portugal).

The important role of cephalopods (Mollusca: Cephalopoda) in many marine ecosystems has been widely acknowledged (Boyle and Rodhouse, 2005). They are commercially exploited around the World (Rodhouse et al., 2014; Arkhipkin et al., 2015; Doubleday et al., 2016; Arkhipkin et al., 2021; Sauer et al., 2021), are predators on numerous prey and are preyed by predators (Santos et al., 2001; Boyle and Rodhouse, 2005; Bello et al., 2011; Hoving et al., 2014; Xavier and Cherel, 2021), whose predator-prey interactions has been helping the development of a conservation framework for some of these predators (Luna et al., 2021). As cephalopod flesh gets quickly digested in predator stomachs, cephalopod beaks (synonym of jaws and mandibles) can resist digestion for as long as several months (Xavier et al., 2005; Barrett et al., 2007). Malcolm Clarke revolutionised the way cephalopod beaks could be used in ecological research, by providing evidence that many of them have unique shapes at species level (Clarke, 1962; Clarke, 1980; Clarke, 1986). Clarke and colleagues also developed the currently used terminology for different parts of the upper and lower beaks (Clarke, 1986) (Figure 1). Such initial efforts helped many other colleagues to develop beak identification guides (Imber, 1978; Pérez-Gándaras, 1983; Wolff, 1984; Kubodera and Furuhashi, 1987; Lu and Ickeringill, 2002; Xavier and Cherel, 2021; Pedà et al., 2022) and supported studies to understand the importance of cephalopods in the diet of different predator taxa (Clarke, 1996; Croxall and Prince, 1996; Klages, 1996; Smale, 1996; Cherel and Klages, 1998; Ménard et al., 2013; Abreu et al., 2019; Romanov et al., 2020; Queirós et al., 2021b; Cherel, 2021; Guímaro et al., 2021). This is particularly important as much information cannot be obtained by other means (e.g., scientific nets are too slow to catch faster cephalopods and catch far fewer species and narrower range of sampled sizes) (Clarke, 1977; Santos et al., 2001; Staudinger et al., 2013; Hoving et al., 2014; Cherel, 2020). Cephalopod beaks can also provide considerable information on a wide range of physiological, biological and ecological traits, including cephalopod availability, consumption of cephalopods, migrations, competition between cephalopod predators, levels of cephalopod scavenging by predators, distribution, age, growth, cohorts, life-events, stress, thermal changes, reproduction, feeding ecology, behavior, spawning areas, post-spawning mortality and sexual dimorphism [e.g., see review in Xavier et al. (2016) and Arkhipkin et al. (2018); Table 1]. More recently, new emergent techniques for work on beaks (e.g., stable isotope and trace elements analyses, geometric morphometrics and microstructure analysis) have provided further information on habitat and trophic position, composition, contamination, response of cephalopods to climate variability at individual and/or population levels, embryonic morphogenesis, paralarval ontogeny, ecology and age estimation (Cherel and Hobson, 2005; Perales-Raya et al., 2014b; Franco-Santos and Vidal, 2014; Xavier et al., 2016; Perales-Raya et al., 2018; Queirós et al., 2018; Golikov et al., 2019a; Golikov et al., 2019b; Northern et al., 2019; Abreu et al., 2020; Queirós et al., 2020a; Armelloni et al., 2020; Queirós et al., 2020b; Franco-Santos and Vidal, 2020; Golikov et al., 2020; Perales-Raya et al., 2020; Fang et al., 2021b; Lishchenko and Jones, 2021). Consequently, the importance of cephalopod beaks in ecological studies continues to attract attention and recognition, with various workshops being organised (Clarke, 1986; Xavier et al., 2007a; Jackson et al., 2007; Xavier et al., 2015). A workshop on cephalopod hard structures including beaks was held in Florida (United States), prior to the 2018 Cephalopod International Advisory Council (CIAC) Conference, results of which should be published in the near future. Most recently, a workshop focused on cephalopod beaks was held in Sesimbra (Portugal) at the 2022 CIAC Conference (Figures A1, A2) in order to review the latest scientific advances on the use of cephalopod beaks in marine ecological studies and discuss future challenges in this and other related research fields. The individual sections below concern key topics in cephalopod research based on the beak analysis discussed during this 2022 CIAC workshop.

**TABLE 1:** A non-exhaustive overview of the use of cephalopod beaks in ecological studies and related research.

Research field	Taxa	References
Taxonomy (including beaks ID) and biogeography	Cephalopoda	Clarke (1962), Akimushkin (1965), Mangold and Fioroni (1966), Iverson and Pinkas (1971), Pinkas et al. (1971), Hotta (1973), Imber (1978), Wolff and Wormuth (1979), Clarke (1980), Wolff (1982a), Wolff (1982b), Pérez-Gándaras (1983), Wolff (1984), Clarke (1986), Kubodera and Furuhashi (1987), Lu and Ickeringill (2002), Franco-Santos and Vidal (2014), Fang et al. (2018), Cherel (2020), Acuña-Perales et al. (2020), Pacheco-Ovando et al. (2021), Xavier and Cherel (2021)
Distribution	Cephalopoda	Clarke (1962), Akimushkin (1965), Mangold and Fioroni (1966), Iverson and Pinkas (1971), Pinkas et al. (1971), Hotta (1973), Clarke (1980), Pérez-Gándaras (1983), Clarke (1986), Kubodera and Furuhashi (1987), Lu and Ickeringill (2002), Xavier et al. (2002b), Xavier et al. (2003a), Cherel et al. (2004), Xavier et al. (2005), Xavier et al. (2006), Xavier and Croxall (2007), Cherel et al. (2009b), Cherel et al. (2011), Xavier et al. (2014), Seco et al. (2016), Pereira et al. (2017), Queirós et al. (2019), Cherel (2020), Guímaro et al. (2021), Queirós et al. (2021a), Xavier and Cherel (2021)
	Squid	Wolff and Wormuth (1979), Wolff (1982a), Wolff (1982b), Wolff (1984), Xavier et al. (2002a), Xavier and Croxall (2007), Cherel et al. (2008), Xavier et al. (2013), Guerreiro et al. (2015), Liu et al. (2015), Queirós et al. (2018), Abreu et al. (2019), Abreu et al. (2020), Cherel (2020), Woods et al. (2022)
	Octopods	Queirós et al. (2020b)
	Bobtail squid	Golikov et al. (2020)
	*Vampyroteuthis*	Golikov et al. (2019a)
Predators vs nets catch composition	Cephalopoda	Clarke (1977), Cherel (2020)
Allometric equations	Cephalopoda	Clarke (1962), Clarke (1980), Clarke (1986), Roeleveld (2000), Lu and Ickeringill (2002), Açik and Salman (2010), Xavier and Cherel (2021)
	Squid	Wolff (1982a), Wolff (1982b), Wolff (1984), Ivanovic and Brunetti (1997), Golikov et al. (2018)
	Octopods	Smale et al. (1993), Golikov et al. (2022)
	*Vampyroteuthis*	Golikov et al. (2019a)
Top predators: in general	Cephalopoda	Clarke (1962), Clarke (1980), Clarke (1986), Santos et al. (2001), Clarke et al. (2002), Ménard et al. (2013), Staudinger et al. (2013), Cherel (2020), Xavier and Cherel (2021)
	Squid	Woods et al. (2022)
Top predators: marine mammals	Cephalopoda	Akimushkin (1955), Clarke (1962), Akimushkin (1965), Clarke (1980), Clarke (1986), Klages (1996), Santos et al. (2001), Pedà et al. (2015), Cherel (2021)
	Squid	Cherel et al. (2008), Abreu et al. (2019)
Top predators: seabirds	Cephalopoda	Pinkas et al. (1971), Furness et al. (1984), Croxall and Prince 1996, Cherel and Klages 1998, Xavier et al. (2003a), Xavier et al. (2005), Xavier et al. (2006), Xavier et al. (2007b), Xavier et al. (2014), Seco et al. (2016), Queirós et al. (2019), Guímaro et al. (2021), Xavier and Cherel (2021)
	Squid	Cherel and Weimerskirch (1999), Xavier et al. (2002a), Xavier and Croxall (2007), Xavier et al. (2013), Guerreiro et al. (2015), Cherel et al. (2017)
Top predators: sharks and other fishes	Cephalopoda	Pinkas et al. (1971), Clarke and Stevens (1974), Smale (1996), Xavier et al. (2002b), Peristeraki et al. (2005), Lansdell and Young (2007), Romeo et al. (2011), Kousteni et al. (2018), Ménard et al. (2013), Romanov et al. (2020), Queirós et al. (2021a)
Global cephalopod biomass estimation	Cephalopoda	Clarke (1977), Clarke et al. (2002)
Community trophic structure	Cephalopoda	Cherel and Hobson (2005), Jackson et al. (2007), Cherel et al. (2009b), Cherel et al. (2011), Guímaro et al. (2021), Queirós et al. (2021a), Queirós et al. (2021b)
	Squid	Guerreiro et al. (2015), Cherel et al. (2019), Abreu et al. (2020), Woods et al. (2022)
	Octopods	Matias et al. (2019), Fang et al. (2021a)
	Bobtail squid	Golikov et al. (2020)
Long-term changes in community structure	Cephalopoda	Guímaro et al. (2021)
	Squid	Abreu et al. (2020)
Trophic ecology of single species	Squid	Castro and Hernández-Garcia (1995), Ruiz-Cooley et al. (2006), Guerra et al. (2010), Fang et al. (2016a), Gong et al. (2018), Liu et al. (2018), Queirós et al. (2018), Trasviña-Carrillo et al. (2018), Hu et al. (2019), Liu et al. (2019a), Liu et al. (2019b), Queirós et al. (2019), Gong et al. (2020), Wang et al. (2022)
	Octopods	Franco-Santos et al. (2014), Queirós et al. (2020b)
	Bobtail squid	Golikov et al. (2019b)
	*Vampyroteuthis*	Golikov et al. (2019a)
Marine trace metal pollution	Squid	Xavier et al. (2016), Northern et al. (2019), Queirós et al. (2020a)
	Octopods	Matias et al. (2020)
Physical and chemical properties of beak material	Cephalopoda	Dilly and Nixon (1976), Uyeno and Kier (2005)
	Squid	Miserez et al. (2007), Miserez et al. (2008), Miserez et al. (2010), Queirós et al. (2018)
	Octopods	Matias et al. (2019)
Migrations	Squid	Cherel and Weimerskirch (1999), Guerra et al. (2010), Liu et al. (2019a), Liu et al. (2019b), Queirós et al. (2019), Queirós et al. (2021a)
Inter- and intraspecific competition	Squid	Gong et al. (2018), Gong et al. (2020)
	Octopods	Matias et al. (2019), Fang et al. (2021a)
	Bobtail squid	Golikov et al. (2020)
Age and growth	Cephalopoda	Bello (1991), Clarke (1993), Arkhipkin et al. (2018)
	Squid	Clarke (1965), Jarre et al. (1991), Liu et al. (2015), Fang et al. (2016a), Hu et al. (2016), Liu et al. (2017), Jin et al. (2019), Perales-Raya et al. (2020)
	Octopods	Perales-Raya and Hernández-González (1998), Hernández-López et al. (2001), Perales-Raya et al. (2010), Rodríguez-Domínguez et al. (2013), Perales-Raya et al. (2014a), Franco-Santos et al. (2016), Garrido et al. (2016), Perales-Raya et al. (2018), García-Fernández et al. (2019), Schwarz (2019), Armelloni et al. (2020), Schwarz et al. (2020)
	*Vampyroteuthis*	Schwarz et al. (2020)
Stock assessment	Squid	Fang et al. (2016a)
Population dynamics	Squid	Liu et al. (2015), Hu et al. (2016), Fang et al. (2021b)
Reproduction	Squid	Hernández-Garcia et al. (1998b), Cherel and Weimerskirch (1999), Xavier and Croxall (2007)
Sexual dimorphism	Squid	Jackson (1995), Bolstad (2006), Cherel et al. (2009a)
Fisheries management	Cephalopoda	Xavier et al. (2007b)
Paleontology	Cephalopoda	Clarke and Maddock (1988)

## Advances in taxonomy, beak morphology, microstructure and paleontology

### Taxonomy and beak morphology

Taxonomic identification is a critical issue of every investigation using accumulated cephalopod beaks from food samples (Table 1). Erroneous identifications can propagate along the studies through the years spreading and proliferating information, not only on predator-prey relationships but also on every subsequent analysis on beaks, regardless the nature of the analyses (e.g., species occurrence/distribution, stable isotopes, trace elements, growth increments) (Cherel, 2020, 2021; Xavier and Cherel, 2021). Substantial efforts have been directed to facilitate the identification of these hard structures through drawings, photographs (from different angles), 3-D videos, measurements, and by materials being accessible through regular publications or on the internet [e.g., Tree of Life web project–http://tolweb.org/articles/?article_id=5274 (Young, 2009); https://www.kahaku.go.jp/research/db/zoology/Beak-E/intro.htm (Kubodera et al., 2005)] (Clarke, 1986; Cherel, 2020; Xavier and Cherel, 2021). Nevertheless, the need for more research experts (e.g., boost/support a new generation of early career scientists in this field) on cephalopod beaks, as well as updated collections and more comprehensive guides will continue to be a necessity in the future (Xavier et al., 2007a; Xavier et al., 2015; Cherel, 2020; Xavier and Cherel, 2021).

Several methods applicable to cephalopod beak shape analysis were developed to date (Fang et al., 2018; Lishchenko and Jones, 2021). Nowadays, the group of methods which can be called “traditional morphometrics” (measurements of the linear distances, or indices, based on these measurements), is criticised for leading to a significant loss of information due to the complexity of studied structures and the multicollinearity between measurements (Adams et al., 2004; Volpedo and Vaz-dos-Santos, 2015). To some extent, this criticism is justified, although these analyses allowed the development of the first steps of beak shape analysis relevant for identification (Mangold and Fioroni, 1966; Wolff, 1982b; Wolff, 1984; Ogden et al., 1998; Fang et al., 2018). Moreover, they are the least laborious among morphometric methods and the richest in terms of available data for comparison.

An alternative approach is represented by two groups of geometric morphometric methods. The first group is based on obtaining Cartesian coordinates of biologically definable points, also called landmarks (Bookstein, 1991; Cadrin, 2014). In addition, sliding semi-landmarks, which are defined by equidistant points between two landmarks are also used to represent curves and surfaces of structures (Bookstein, 1997; Gunz et al., 2005; Gunz and Mitteroecker, 2013). These landmarks and semi-landmarks may be two- or three-dimensional, and generally are discrete and homologous (Zelditch et al., 2004). The coordinates obtained are modified using Procrustes analysis to avoid impact of the position and size of the studied object.

The second group of methods includes those methods which describe the structure’s outline as a whole. The most applied methods in this group are Fourier transform (where outlines are expressed as a function of equally spaced radii or of the tangent angle to the outline or of the curvilinear abscissa) and wavelet transform (with outlines expressed by a set of functions representing the dilations and translations of a single unique function). Application of both approaches to analyse the shape of cephalopod beaks have benefits and limitations (Lishchenko and Jones, 2021). Specifically, landmark-based methods allow selecting the points of interest which presumably have some biological or taxonomic meaning (e.g., rostral tip and wing fold, whose position reflects the length and curvature of the rostrum). On another hand, landmark selection inevitably leads to the loss of information, which may be crucial if the points of interest were chosen incorrectly. Additionally, process of landmark selection is particularly laborious, time consuming and demands a certain level of qualification of the researcher. The outline-based methods of the shape analysis do not have these drawbacks and allow detailed description of a beak’s contour on the image. However, the latter methods are associated with a very specific issue, which is called a “pixel noise” by some authors (Haines and Crampton, 2000; Harbitz and Albert, 2015) (i.e., “pixel noise” stands for the excessive and meaningless set of information which hampers the analysis of the structure’s shape).

Despite these limitations, the potential of these approaches is substantial, supported by the results of recent studies. Most commonly, geometric morphometrics methods were successfully applied for taxonomic classification, species identification or stocks (Neige and Dommergues, 2002; Crespi-Abril et al., 2010; Tanabe et al., 2015b; Fang et al., 2017; Jin et al., 2017; Fang et al., 2018; Pacheco-Ovando et al., 2021; Díaz-Santana-Iturrios et al., 2022). At the beginning of the millennium, the application of these approaches to a wide diversity of species was scarce (Neige and Dommergues, 2002; Tanabe et al., 2015b) and provided limited resolution of identification. Their findings suggested that the 2-D lateral shapes of beaks (lateral wall, hood and wing contours) are clustered at high taxonomic levels (orders, suborders) and that the upper and lower parts of the beak carry a slightly different information. By showing that to be true, quantitative analysis of beak shape might assist identification at high taxonomic levels (at least, to the level of family). However, in the last decade, geometric morphometric methods began to flourish. Different authors applied either 2D landmark-based methods (Fang et al., 2017; Fang et al., 2018; Pacheco-Ovando et al., 2021; Díaz-Santana-Iturrios et al., 2022) or outline-based methods (Jin et al., 2017). Both approaches showed high level of identification accuracy, up to 100% in classification of the genera (Pacheco-Ovando et al., 2021), up to 93% in species identification (Fang et al., 2018), and up to 70% correct classifications of stock units (Fang et al., 2017). These results point to the potential to engineer automated identification programs.

Several studies revealed the potential of beak shape analysis in ecological studies (Fernández-Álvarez et al., 2020; Pacheco-Ovando et al., 2021; Roscian et al., 2022). Specifically, Fernández-Álvarez et al. (2020) studied impacts of developmental malformations of the buccal mass on the trophic position of *Eledone cirrhosa* and found that the habitat and the trophic position were not significantly affected by the malformations. Other authors found significant differences in the beak shapes of pelagic and benthic species in relation to their trophic levels (Roscian et al., 2022), between species living in coastal and oceanic habitats (Pacheco-Ovando et al., 2021) and between species/populations living in different feeding habitats (Pacheco-Ovando et al., 2021). New phylogenomic techniques applied to cephalopods (Anderson and Lindgren, 2021; Sanchez et al., 2021; Fernández-Álvarez et al., 2022) may help to assess which morphological characters of the beaks are determined by phylogeny and which are explained by other drivers.

Geometric morphometric studies of cephalopod beaks have the greatest potential in the field of species identification, as part of both the routine monitoring process and as high-end studies. Application of outline-based methods allows it even without additional efforts. At present, an automated similar system has been used for the identification of fish using otolith contours (Lombarte et al., 2006). This approach shows that this system may allow accurate identification of animals even when there is only very basic information available about the subject of research and it is probably the least time-consuming method of them all. Development of such a system is a long-term process that needs close validation checks, but even at the early stage of development, it could substantially expand our knowledge on cephalopods.

### Beak morphological changes during the early life

**FIGURE 2:**
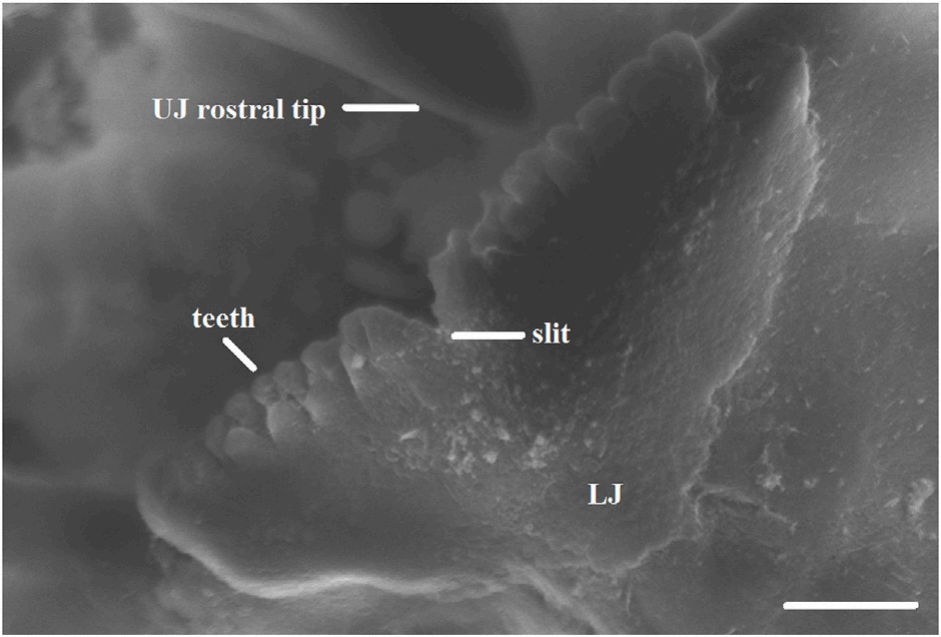
Close-up of a scanning electron microscope image indicating (lines) the teeth and slit in the lower beak (LJ) and the rostral tip in the upper beak (UJ) of an *Illex argentinus* paralarva of 4.0 mm ML. Scale bar 10 μm ((Franco-Santos and Vidal, 2020); copyright permission obtained).

Beaks of embryos and paralarvae are quite distinct from those of juveniles and adults (Franco-Santos et al., 2014; Franco-Santos and Vidal, 2014; Franco-Santos et al., 2016; Armelloni et al., 2020; Franco-Santos and Vidal, 2020). The beaks of hatchlings and smaller squid and octopus paralarvae studied so far, are very fragile and slightly pigmented, being nearly transparent. Growth rings might or not be clearly visible in the lateral wall of both the upper and lower beaks (Franco-Santos and Vidal, 2014), and they might be visible in the anterior pigmented region of upper beaks that corresponds to the rostrum (Perales-Raya et al., 2014b; Franco-Santos et al., 2016; Arkhipkin et al., 2018; Perales-Raya et al., 2018). Paralarval beaks in several cephalopod families (E.g., Family Ommastrephidae, family Octopodidae) have teeth that might be present in both beaks or only in the lower one (Boletzky, 1971; Wakabayashi et al., 2002; Uchikawa et al., 2009; Franco-Santos et al., 2014; Franco-Santos and Vidal, 2014; Franco-Santos and Vidal, 2020). In the upper beak of many species, the rostrum has not yet protruded, and has a typical sagittal slit between the two-halves that could be slightly or very pronounced (Figure 2). In addition, conspicuous features of the beaks, such as the hood and lateral walls might not be developed yet in paralarvae of some families, such as Ommastrephidae (Franco-Santos and Vidal, 2020), giving the beak a more rounded shape. A study with late embryonic hatching stages of *Octopus vulgaris* embryos has shown that the upper beak is rudimentary and lacks the hood, but the teeth are already visible and these stages represent the beginning of the pigmentation process. The hood and shoulder develop along with the exposure of the dentition on the rostrum just prior to hatching (Armelloni et al., 2020), suggesting that beak development is intensified afterwards during the paralarval phase (Franco-Santos and Vidal, 2014; Franco-Santos and Vidal, 2020).

The beak morphology of smaller paralarvae suggests a weak bite force. Thus, at this stage the beak has not yet assumed the functions of biting flesh and masticating the hard exoskeleton of crustaceans (Franco-Santos and Vidal, 2014). Indeed, beaks of smaller paralarvae seem to be adapted for a specialised feeding mode that involves external pre-digestion and suction of body fluids of crustacean prey (Franco-Santos et al., 2014; Franco-Santos and Vidal, 2014; Franco-Santos and Vidal, 2020). This is supported by observations of the feeding behaviour of *O. vulgaris* and loliginid squid paralarvae (Hernández-García et al., 2000; Franco-Santos and Vidal, 2014).

As paralarvae grow, the teeth are eroded and disappear, and the hood and lateral walls grow rapidly as they are important sites for buccal musculature attachment (Uyeno and Kier, 2007). The sagittal slit progressively closes and gives way to the rostrum. The pigmented area increases through the deposition of sclerotized layers (i.e., not chitin during the darkening process), particularly in the rostrum, which protrudes very fast in both beaks (Franco-Santos and Vidal, 2014; Franco-Santos and Vidal, 2020). Beak darkening is a continuous process, through which beaks become harder and more robust (Hernández-García et al., 1998a, b; Miserez et al., 2007). Fully pigmented beaks were considered to be “mature beaks” by Clarke (1986).

These studies have shown that the underdeveloped beaks of paralarvae are rapidly transformed, giving way to the prominent structures present in juvenile and adult beaks. Such studies have provided the foundation for inferences about rostrum functionality and its relationship with feeding strategy and prey selection during the first stages of the life cycle, about which little is known (Franco-Santos and Vidal, 2014; Nande et al., 2017; Vidal and Salvador, 2019; Franco-Santos and Vidal, 2020). In addition, beaks of *O. vulgaris* paralarvae have been used to validate daily growth increments in the early stages, for comparison of wild and cultured specimens to understand massive mortalities previous to the juvenile phase (Garrido et al., 2016; García-Fernández et al., 2019) and to solidify our understanding of the significant influence of temperature on increment deposition (Perales-Raya et al., 2018). The accuracy of age estimation inferred from growth marks in the upper beak rostrum has been also confirmed in late-stage embryos (Armelloni et al., 2020). In addition, it was suggested that these growth marks might be used as biomarkers for stress during rearing of *O. vulgaris* paralarvae (Franco-Santos et al., 2016).

### Microstructure: Age, growth and record of life extreme events

**FIGURE 3:**
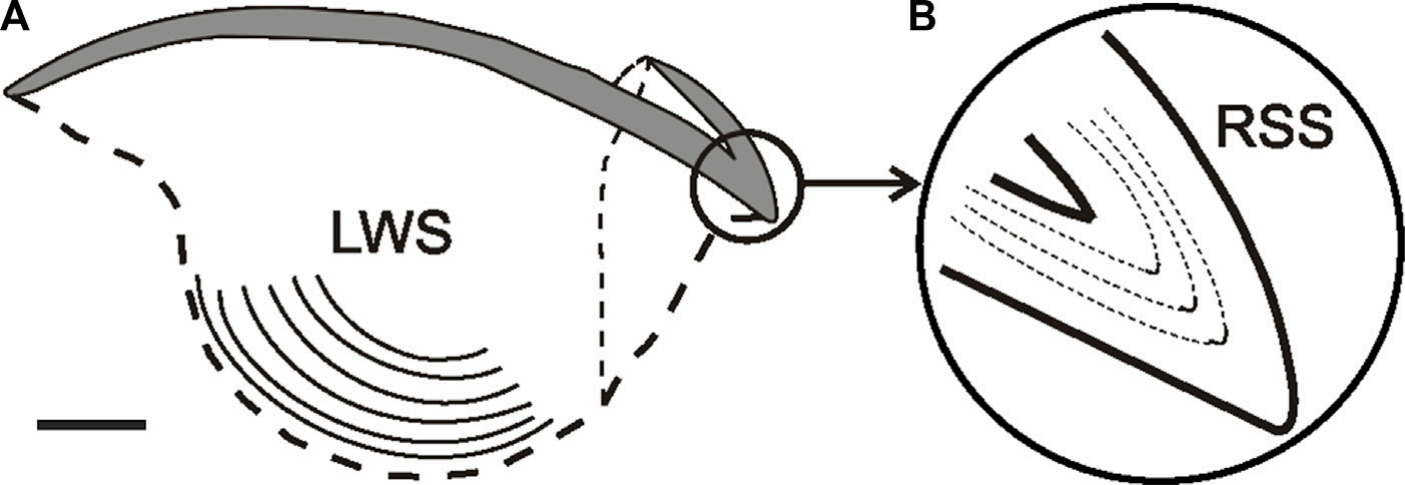
Lateral view of the upper beak in *Octopus vulgaris* **(A)** Sagittal section showing the inner lateral wall surface (LWS) bearing increments (lines). **(B)** Rostrum sagittal section magnified and showing the daily increments. From Perales-Raya et al. (2014a); copyright permission obtained. Bar: approx. 2 mm.

Although statoliths are the most frequently used material for age determination in cephalopods, it has been suggested that beaks could provide additional/complementary data, especially when it is impossible to obtain access to age data from statoliths (Liu et al., 2014). This is the case for octopods, which lack visible increments in the microstructure of their statoliths (Clarke, 1978). In addition, statoliths of adult cephalopods need to be ground on both sides, which is time-consuming and labour-intensive, whereas beaks do not need to be ground on both sides (Arkhipkin et al., 2018). Beaks are present throughout the life cycle of all extant cephalopod species, and can be easily extracted and preserved (Clarke, 1986). Beak growth process takes place along the posterior border, where the most recent chitinized and hydrated material is deposited, so that the oldest and most pigmented material is found in the anterior tip (Miserez et al., 2008; Perales-Raya et al., 2014a). Moreover, growth increments have been observed and validated along several parts of cephalopod beaks [e.g., lateral wall surfaces, rostrum sagittal sections (Perales-Raya et al., 2010)]. Perales-Raya et al. (2010) recommended counting growth increments in the lateral wall surface of beaks of *Octopus vulgaris* as fewer increments were detected in rostrum sagittal sections, probably due to erosion of the rostral tip when the animal is feeding. In some cephalopods (including *O. vulgaris*) these increments have been validated as being deposited daily (see below for further discussion). Thus, counts of growth increments on these structures can potentially provide absolute age estimates and growth data in any ontogenetic phase (Figure 3). Comparison with other structures in which deposition of growth increments is thought to be daily, as it has been validated for statoliths in several species of squid (E.g., *Illex illecebrosus, Loliolus noctiluca, Loligo chinensis, Loligo vulgaris reynaudii*) and cuttlefish (E.g., *Sepia officinalis*) (Hurley et al., 1985; Jackson, 1990; Lipinski et al., 1998; Bettencourt and Guerra, 2001), can be used to infer the periodicity of increment deposition in the beaks of newly studied species or when validation experiments are not feasible. Nevertheless, validation experiments involving mark-recapture or captive rearing of known-age or chemically-marked specimens are needed to ensure absolute age determination in the species (Campana, 2001). Even then, and considering that a circadian rhythm has been proved in many species (Cobb et al., 1995; Meisel et al., 2003), the fact that growth increments are shown to be deposited daily does not prove that this will always be the case to all cephalopod species/populations as increment deposition may depend on various factors (e.g., food availability, water temperature) (Bettencourt and Guerra, 2000; Zumholz et al., 2006; Canali et al., 2011).

Since growth increments in beaks were first reported in the 1960s for the squid *Moroteuthopsis longimana* (misidentified as *Moroteuthis ingens* by Clarke (Clarke, 1965; Cherel, 2020), many attempts have been made to use these structures to estimate cephalopod age (Tables 1, 2). Indeed, the growth increments in cephalopod beaks, initially observed by Clarke (1965), in the surface of lateral walls did not show a suitable sequence of increments to estimate the age in the species examined. The use of beak microstructure for age estimation was re-assessed in the 1990’s in octopuses (Perales-Raya and Hernández-González, 1998), due to the absence of evident increments in their statoliths. Perales-Raya and Hernández-González (1998) observed a sequence of thin increments in sagittal sections of the rostrum of *O. vulgaris* beaks, and suggested that their deposition should be related to an individual’s age. Successful analysis of lateral wall surfaces of *O. vulgaris* beaks was then performed by Hernández-López et al. (2001) and daily deposition was confirmed in paralarvae. Then, both techniques were compared and improved by Perales-Raya *et al.* (2010). Validation studies were subsequently performed to confirm daily deposition also in octopuses (E.g., *Octopus vulgaris*, *Octopus maya*) (Canali et al., 2011; Rodríguez-Domínguez et al., 2013; Bárcenas et al., 2014). Finally, daily deposition was validated in the full ontogenetic range for *O. vulgaris* on both the rostrum sagittal sections and the lateral wall surfaces by Perales-Raya *et al.* (2014a). These authors used the recording of specific events in beaks of *O. vulgaris* taken into captivity (e.g., capture, temperature changes) as dated marks to validate the temporal deposition of increments. Consequently, the analysis of beaks as life event recorders has been applied in wild populations to understand the effects of environmental variations, biological events, stress of capture (Perales-Raya et al., 2014b), and to improve the welfare of early stages in reared populations (Franco-Santos et al., 2016).

**TABLE 2:** Detailed information on cephalopod species that have been attempted using beak increment analysis.

Species	Beak part	Validated	Study
Octopoda and Vampyromorpha			
*Octopus vulgaris*	Rostrum sagittal section of upper beak	No	Perales-Raya and Hernández-González, (1998)
	Lateral wall of upper beak	Yes (paralarvae)	Hernández-López et al. (2001)
	Rostrum of upper beak	Partially (5 specimens marked)	Oosthuizen, (2003)
	Rostrum sagittal section of upper and lower beak; lateral wall of upper beak	No	Perales-Raya et al. (2010)
	Lateral wall of upper beak	Yes	Canali et al. (2011)
	Lateral wall of upper beak	No	Castanhari and Tomás, (2012)
	Lateral wall of upper beak	No	Cuccu et al. (2013)
	Lateral wall and rostrum sagittal section of upper beak	No	Perales-Raya et al. (2014b)
	Lateral wall and rostrum sagittal section of upper beak	Yes	Perales-Raya et al. (2014a)
*Octopus maya*	Rostrum sagittal section of upper beak	Yes	Bárcenas et al. (2014)
	Lateral wall of upper beak	Yes	Rodríguez-Domínguez et al. (2013)
*Octopus huttoni*	Lateral wall of upper beak	No	Donlon et al. (2019)
*Pareledone aequipapillae, Pareledone charcoti, Megaleledone setebos, Muusoctopus rigbyae, Adelieledone polymorpha, Pareledone aurata, Pareledone felix, Pareledone turqueti*	Lateral wall of upper beak	No	Schwarz et al. (2019)
*Japetella diaphana, Vampyroteuthis infernalis*	Lateral wall of upper beak	No	Schwarz et al. (2020)
Cuttlefish			
*Sepia apama*	Lateral wall of upper beak, rostrum of upper beak	No	Hall, (2002)
Squid			
*Illex argentinus*	Lateral wall of upper beak	Yes (paralarvae)	Sakai et al. (2007)
	Rostrum sagittal section of upper beak	No (cross-verification with statoliths)	Liu et al. (2015)
*Ommastrephes caroli*	Lateral wall of upper beak	Yes (paralarvae)	Sakai et al. (2007)
	Rostrum sagittal section of upper beak	No (cross-verification with statoliths)	Liu et al. (2015)
*Dosidicus gigas*	Lateral wall of upper beak	Yes (paralarvae)	Sakai et al. (2007)
	Rostrum sagittal section of upper beak	No (cross-verification with statoliths)	Liu et al. (2015)
*Sthenoteuthis oualaniensis*	Lateral wall of upper beak	Yes (paralarvae)	Sakai et al. (2007)
	Rostrum sagittal section of upper beak	No (cross-verification with statoliths)	Liu et al. (2015)
*Todarodes pacificus*	Lateral wall of upper beak	Yes (paralarvae)	Sakai et al. (2007)
*Architeuthis dux*	Rostrum sagittal section of lower beak	No	Perales-Raya et al. (2020)
*Uroteuthis chinensis*	Rostrum sagittal section of upper beak	No	Jin et al. (2019)
*Uroteuthis edulis*	Rostrum sagittal section of upper beak	No	Lin et al. (2019)
*Histioteuthis bonnellii*	Lateral wall of upper beak	No	Mereu et al. (2011)

**FIGURE 4:**
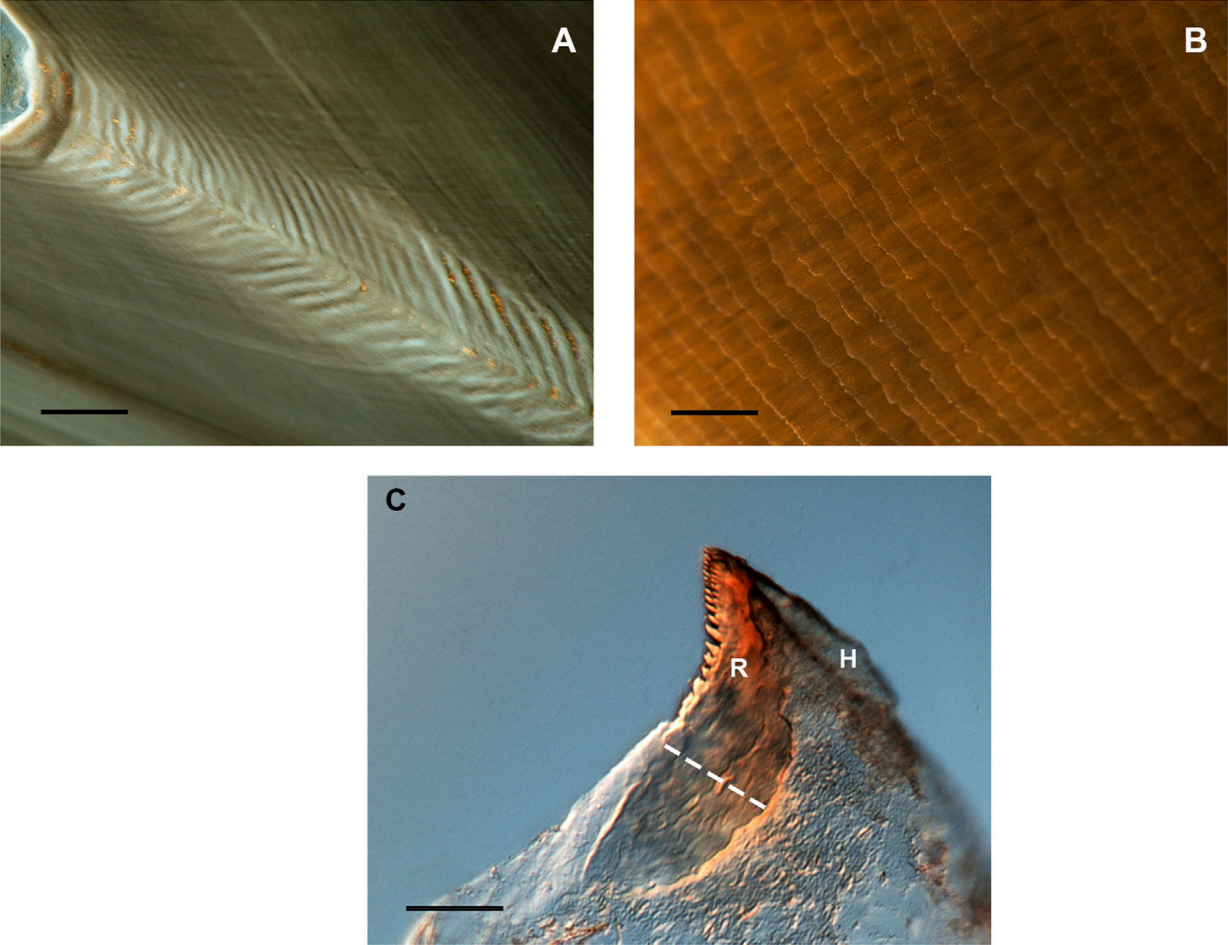
Microstructure of beaks showing growth increments in cephalopod beaks (*Octopus vulgaris*). **(A)** Increments in the rostrum sagittal sections (RSS). Bar: 100 µm. **(B)** Increments in the inner lateral wall surface of the upper beak. Bar: 200 µm. **(C)** Increments in the rostrum surface of the upper beak in early stages. R *=* rostrum; H *=* hood; dotted white line = reading area. From Arkhipkin et al. (2018) and Perales-Raya et al. (2018); copyright permission obtained). Bar: 50 µm.

Since the daily deposition was validated in *O. vulgaris* (Figure 4), the beak microstructure has been used in age and growth studies of several species of squid, cuttlefish and octopods, providing key information on their life history and population dynamics (Cuccu et al., 2013; Fang et al., 2016a; Liu et al., 2017; Batista et al., 2021). Validation studies have been performed in species such as *Sepia officinalis* (Lishchenko, unpublished data) and *Octopus insularis*, based on the growth increments in the rostrum sagittal sections or lateral wall surface of the beak (see references in Table 2).

**FIGURE 5:**
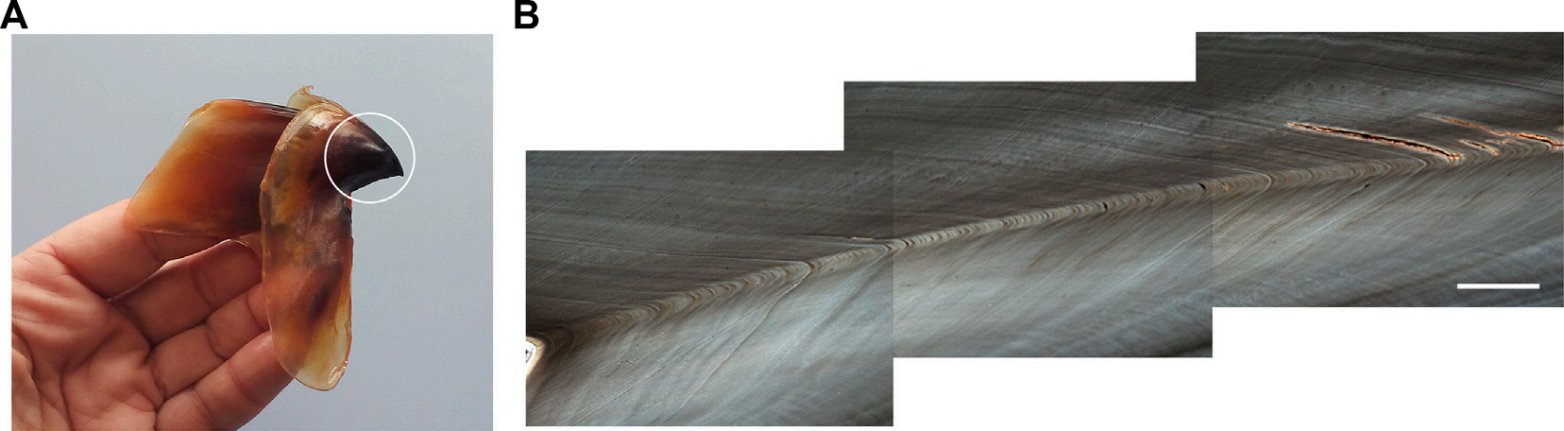
Lower beak of the giant squid *Architeuthis dux*. A white circle highlights the rostrum **(A)**. Image composition of the sagittal section of the lower beak rostrum showing growth increments **(B)**. From Perales-Raya et al. (2020); copyright permission obtained). Bar: 200 µm.

In relation to using beaks for age determination in cephalopods, it was found that the tip erosion during the feeding process may bias increment counts in the anterior region of the beak but counting the oldest increments in the dorsal area of the section prevents age underestimation (Arkhipkin et al., 2018). A simple method has been recently developed to quantify the tip erosion using the number of increments in the dorsal non-eroded region and the width of increment of the central reading region (Perales-Raya et al., 2020). Moreover, the beak microstructure of emblematic deep-sea species such as the giant squid *Architeuthis dux* (Perales-Raya et al., 2020) (Figure 5) provided age data and a maximum lifespan estimation of around 3 years, based on rostrum sagittal sections from the lower beaks. The same technique was used in the beaks of the warty squid *M. longimana* from the stomach contents of Antarctic toothfish to estimate the age of this Southern Ocean species (Queirós, Bartolomé, Xavier, Perales-Raya, unpublished data). In both studies, the authors tested the lateral wall surface of upper beaks and the rostrum sagittal sections of upper and lower beaks but only the latter showed a suitable sequence of increments for age estimation. On the contrary, the lateral wall surface has been the only beak region explored in Antarctic incirrate octopods from the families Megaleledonidae and Enteroctopodidae (Schwarz, 2019; Schwarz et al., 2019). These authors suggested lifespans exceeding 3 years and the possibility that deposition of growth increments in beaks of Antarctic octopods is not daily.

Growth increments (usually a few dozen) are also known on the surface of ammonite aptychi (calcitic coverings of the lower beak). The major growth increments were hypothesised to represent fortnightly tidal cycles or months, and the minor increments to correspond to days or semi-diurnal tidal cycles. If this assumption is correct, the ammonite life span would last between 1 and 6 years and the growth would be sigmoidal, with slowing down at maturity (Hewitt et al., 1993; Machalski, 2021).

### Palaeontology: Fossil beaks

The nautiloid and ammonoid beaks are the cephalopod structures that fossilise most effectively after the shells. They are most often preserved in 2D but 3D fossils occasionally occur (Nixon, 2015). Ammonoid lower beaks are the most abundant in the fossil record, particularly from the Lower Jurassic (Toarcian) where a bivalve calcareous covering appears on the lower beak (Tanabe et al., 2015a). Similarly, the rostrum of the upper beaks of nautiluses, consisting of a hard, pointed calcareous tip (rhyncholite), is quite common in the fossil record from the Triassic onwards. Entirely organic beaks such as those of coleoids are very rare in the fossil record but are nevertheless known from exceptionally well-preserved deposits, the so-called *Lägersttätten*. Numerous examples are found in ammonoids dating back to the Devonian (Tanabe et al., 2015a; Klug et al., 2016). They are rarer in the Nautilida, but a few specimens are usable for shape analysis (Klug et al., 2021a). In Coleoids, beaks are found from the end of the Lower Jurassic (Nixon, 2015; Klug et al., 2021b). Fairly well-preserved 3D specimens have been related to Sepiolida (Harzhauser, 1999), Oegopsida (Tanabe et al., 2006), Cirrata (Tanabe et al., 2008), Vampyromorphida (Tanabe and Hikida, 2010), or undetermined teuthid taxa (Tanabe et al., 2015b).

All these fossils are still underused because of the shortage of comparative analyses between current and fossil beaks. The identification of beak shape adaptations to habitats or prey selection in modern species could inform our understanding of fossil shapes, thus complementing the classical paleoecological inferences from shells, gladius, soft tissues or fossil deposits (Fuchs and Iba, 2015; Fuchs et al., 2016). For example, investigating habitat shifts from shallow neritic to mesopelagic or bathyal environments (Hoving et al., 2014; Košťák et al., 2021) or *vice versa* (Arkhipkin et al., 2012) is of importance for understanding the evolution of modern coleoid lineages and for interpreting radiation events during cephalopod evolutionary history.

Currently, the data support a significant diversification event in Octopodiformes and Decapodiformes in relation to the so-called Marine Mesozoic Revolution, characterised by a major remodelling of shallow ecosystems, the rise of durophagous predators, and the resulting ecological “arms race” between predators and prey (Salamon et al., 2012). In this context, the diversification of coleoid cephalopods (stem Decabrachia, Spirulida) would have been driven by competition with predatory fish (Packard, 1972; Tanner et al., 2017; López-Córdova et al., 2022). The lineages of modern coleoid species are thought to have originated from one or more radiation pulses before the Cretaceous-Paleogene extinction (K-Pg crisis) (Fuchs and Lukeneder, 2014; López-Córdova et al., 2022). After the K-Pg crisis, a radiation pulse was stimulated by the disappearance of many predators and the availability of ecological space. Finally, another radiation pulse occurred during the mid-Cenozoic, controlled by climate change and low predator pressure (Fuchs and Lukeneder, 2014; López-Córdova et al., 2022). New data contributing to the inference of habitat, trophic level or predatory abilities of fossil species will be useful to assess the proposed evolutionary scenarios. To achieve this goal, thorough analysis of the relationship between beak shape and species ecology in current cephalopods, followed by integration of the results with the fossil species would be an excellent step forward.

## Advances in analysis if the composition of cephalopods beaks relevant to ecological studies

Cephalopod beaks are secreted by a single layer of cells in the buccal tissue, the beccublast cells, which are tall columnar epithelial cells (Dilly and Nixon, 1976; Tan et al., 2015). Beaks grow without replacement throughout the life of cephalopods (Perales-Raya et al., 2014a). Therefore, performing chemical analyses on entire beaks yields an average value for the entire life of the individual, whereas dividing the beak into different sections allows us to study different periods within the life cycle (Cherel and Hobson, 2005; Cherel et al., 2009a; Guerra et al., 2010; Xavier et al., 2016; Queirós et al., 2020a).

### Beak chemical composition as a challenge to the application of chemical analyses

Beaks are composed of chitin-protein complexes (Miserez et al., 2007). There are differences in the composition among cephalopod taxa, with beaks of octopods being composed exclusively of α chitin, whereas those from squid, while mainly composed by α chitin, also contain β chitin (Miserez et al., 2007; Matias et al., 2019). However, these variations are not expected to result in significant differences when performing chemical analyses on these structures. In contrast, the ratio between chitin and protein varies along the beak, which can have significant influences on the analyses (e.g., lowering of *δ* ^15^N values when the amount of chitin is higher) (Miserez et al., 2008; Tan et al., 2015). These differences in the chitin: protein ratio can be easily observed in the pigmentation, with the untanned parts of the beak having a higher content of chitin than the fully tanned portion, which has a higher protein content (Miserez et al., 2008).

### Stable isotopes

Stable isotopes, particularly of *δ* ^13^C and *δ* ^15^N, are widely used in ecological studies (Peterson and Fry, 1987; Bearhop et al., 2004; Newsome et al., 2007). Using *δ* ^13^C values, it is possible to determine the carbon source at the base of the food chain and, ultimately, the feeding habitat of individuals (Cherel and Hobson, 2005). In marine systems, *δ* ^13^C values are known to vary with latitude (lower towards the poles), with inshore–offshore gradient (lower values towards offshore waters) and between benthic and pelagic environments (lower in pelagic organisms) (Cherel and Hobson, 2005; Newsome et al., 2007; Magozzi et al., 2017). In addition, anthropogenic CO_2_ in the atmosphere has resulted in a decrease in *δ* ^13^C (as well as in ^14^C) in both the atmosphere and the oceans, due to fossil fuels being relatively depleted in heavier carbon isotopes, the so-called Suess effect (Keeling, 1979; Gruber et al., 1999; Sonnerup et al., 1999). Regarding *δ* ^15^N values, these are used to study the trophic position of the individuals, based on the principle that predators are enriched in ^15^N in relation to their prey (Peterson and Fry, 1987).

Bulk stable isotopic analyses (SIA) on beaks have been routinely perfomed, with *δ* ^13^C and *δ* ^15^N measured in cephalopods from all ocean basins (Hobson and Cherel, 2006; Cherel et al., 2009b; Guerra et al., 2010; Navarro et al., 2013; Golikov et al., 2019a; Queirós et al., 2020b; Fang et al., 2021a). They have been used: 1) in ecological and biogeographical studies to determine the foraging habitat and the role of cephalopods in food webs (Ruiz-Cooley et al., 2006; Guerra et al., 2010; Golikov et al., 2018; Staudinger et al., 2019; Fang et al., 2021a), 2) in fisheries to study stocks’ distribution, contributing also to the implementation of ecosystem-based management (Fang et al., 2016b; Queirós et al., 2019), and 3) to study impacts of climate change and environmental fluctuations in these organisms (Golikov et al., 2019a; Hu et al., 2019; Abreu et al., 2020). Furthermore, SIA on beaks can also be used to study the foraging ecology of their predators (Guerreiro et al., 2015; Abreu et al., 2019; Guímaro et al., 2021). SIA of the entire beak gives us an average value for the life of the individual, and when applied to different sections of the beak (using the methodology specifically applied for SIA analyses (Guerra et al., 2010; Queirós et al., 2018)), it enables the study of ontogenetic changes throughout the lifespan (Cherel and Hobson, 2005; Guerra et al., 2010; Queirós et al., 2018; Wang et al., 2022). More recently, compound specific stable isotopes on amino acids (CSIA-AA) have also been studied in cephalopod beaks, mainly to delete the chitin effect that lowers the bulk *δ* ^15^N values of beaks when compared to other tissues (Cherel et al., 2019; Woods et al., 2022) (see below).

The SIA of beaks is a well-established technique with several advantages when compared to the use of other tissues. Cephalopod beaks recovered from predators’ stomachs have comparable stable isotopic compositions regardless the time they were subjected to digestive processes and independently of the time-period during which they have been kept in collections (Cherel and Hobson, 2005; Abreu et al., 2020). The preservation method (e.g., dried, frozen, in ethanol, in formalin) also does not affect SI values/ratios, enabling its application to museum beak collections and retrospective investigations (Ruiz-Cooley et al., 2011). However, when using SIA, there are some caveats to be aware of (see those listed below). Some of these can be overcome using CSIA-AA but due to the higher costs of this technique, its wider use has been so far limited. In addition, there are common problems (e.g., amount of sample required, knowledge of the trophic enrichment factor) associated with both CSIA-AA and bulk SIA. Below, we summarise the main problems associated with SIA, how to detect them and possible solutions. First and foremost, the amount of sample (usually ∼0.35 mg but it is dependent of the equipment/methodology/analyzer used) that is necessary for both SIA and CSIA-AA (and other analyses) can be a limitation, especially if the study aims for a sequential analysis along the beak or concerns smaller species/specimens whose entire beaks do not make up the necessary mass for the analyses. In this situation, a possible solution is to pool the minimum number of different beaks necessary to perform the analyses. However, it must be guaranteed that beaks belong to similar individuals (i.e., similar size, collected in the same location and at the same time) to ensure they belong to the same school/cohort (e.g., Guímaro et al., 2021; Queirós et al., 2021a). Interpretation of SIA results from cephalopod beaks (and other tissues) is dependent on baseline stable isotopic values (i.e., to determine the habitat and trophic level). It is advisable to determine the *δ* ^13^C values of Particulate Organic Matter (POM) from the region and/or the *δ* ^15^N values of an organism with a known trophic position (e.g., *δ* ^15^N value of a filter-feeder species that it is in the second trophic level). To overcome the necessity of analysing POM stable isotopic values, it is possible to use values obtained from previous studies, or studies that modelled isotopic variation around the world’s oceans (Somes et al., 2010; Farmer et al., 2021; St John Glew et al., 2021). However, when using values from previous studies or when comparing values obtained in beaks collected in different periods, it is necessary to account for the temporal variation of isotopes in the environment. To solve this, the obtained *δ* ^13^C values need to be corrected considering the Suess effect when values span across decades or consider modelled past *δ* ^13^C values (Farmer et al., 2021). For the *δ* ^15^N, the use of values obtained by previous modelling studies is so far the best option (Somes et al., 2010; Farmer et al., 2021; Verwega et al., 2021). If the capture location of the individual is known, the isotopic value of the near-death beak segment [i.e., the end of the hood and crest in the upper beak and the end of the hood and crest and wing in the lower beak (Queirós et al., 2018)], can be related to the local isotopic values. This may not be appropriate when using beaks sampled from predators’ stomachs, since it needs to also take into consideration the daily (and long-time scale) movements of the predator and the time the beak spent in the stomach and these will vary depending on predator and cephalopod species involved (Xavier et al., 2003b; Xavier and Cherel, 2021). Nevertheless, for bulk SIA, without measuring baselines at the time of collection, it is impossible to estimate the trophic position of the individual. In contrast, a baseline is not necessary when determining the trophic position using CSIA-AA because the use of so-called “source amino acids” (e.g., phenylalanine) provides a baseline for the cephalopod beak (Cherel et al., 2019). However, using bulk SIA it is still possible, by comparing different beaks or different sections of the beaks (Queirós et al., 2018), to identify changes in habitat or trophic position. One limitation when studying changes in habitat is that changes in *δ* ^13^C values can be related to latitudinal, inshore-offshore, or benthic-pelagic changes (Cherel and Hobson, 2007; Newsome et al., 2007); thus results should be considered carefully and conclusions should be supported by previous knowledge of the ecology of the species. Furthermore, it is important to note that *δ* ^13^C also increases by around ∼1‰ per trophic level through the food web, so that a significant correlation between *δ* ^13^C and *δ* ^15^N values could be due to a habitat change or diet change. Determining the slope of a regression between *δ* ^13^C and *δ* ^15^N values may however enable these two possibilities to be distinguished.

Regarding trophic position changes, when comparing *δ* ^15^N values, it is important to know the relevant trophic enrichment factor (TEF) (trophic discrimination factor (TDF) in CSIA-AA) (i.e., the differences between the *δ* ^15^N values of predator and its diet). Previous studies using *S. officinalis* raised in captivity showed that for beaks, this value is ∼3.4‰ (Hobson and Cherel, 2006). However, recent studies showed that the TEF tends to decrease with increasing trophic position, requiring the use of the “scale *δ* ^15^N framework” (Hussey et al., 2014a; b). This *δ* ^15^N framework also needs to be region specific (Hussey et al., 2014a; b). In CSIA-AA, the *δ* ^15^N framework is not applicable and a TDF of ∼7.6‰ is used universally (O’Connell, 2017; Whiteman et al., 2019), although no cephalopod-specific studies exist to date. Also, CSIA-AA requires information on the difference between *δ* ^15^N values of «trophic» and «source amino acids » in producers (trophic level 1), commonly referred to as *β*, to estimate consumer’s trophic position. A value of *β* of −3.4‰ is universally used in aquatic ecosystems (O’Connell, 2017; Whiteman et al., 2019). It should be borne in mind that *δ* ^15^N values do not change only with the trophic position or prey species, but can be related to other changes in the diet of the predators such as the proportions of different prey that are eaten (Bearhop et al., 2004) and to changes in the diet of the prey species.

Currently neither bulk nor compound-specific SIA allows the identification of specific prey species. Even if the measurement of *δ* ^13^C and *δ* ^15^N values in a specific target prey species is performed, it is difficult to confirm that the tested species was eaten rather than another species with similar diet and trophic level (Fogel and Tuross, 2003; Naito et al., 2013; Pinkerton et al., 2014; Golikov et al., 2020). So-called mixed models are commonly used to infer predator diet composition from bulk SIA data. They require information on the *δ* ^13^C and *δ* ^15^N values of all putative prey but, even then, the natural variation around average values needs to be accounted for and, fundamentally, using values of only two variables (*δ* ^13^C and *δ* ^15^N values) to determine the values of multiple model parameters (the dietary importance of *N* putative prey types) is difficult (Phillips et al., 2014). In this sense, CSIA-AA offers a way forward since *δ* ^13^C and *δ* ^15^N values are potentially available for multiple trophic amino acids, greatly increasing the theoretical discriminatory power of the data to allow diet composition to be determined.

The *δ* ^13^C and *δ* ^15^N values obtained from bulk SIA and CSIA-AA are not directly comparable, and even if using the estimated trophic position allows the two types of *δ* ^15^N measurement to be compared indirectly, there is currently no such method for *δ* ^13^C. One key limitation when applying SIA to beaks is their chitin content. Chitin is a polymer of N-acetyl-glucosamine (i.e., a complex sugar that contains N atoms that are impoverished in ^15^N when compared to amino acids). Thus, *δ* ^15^N values are not only lower in beaks than in other tissues, but also vary between different sections of the same beak due to the varying chitin: protein ratio (as mentioned above). It is necessary to carefully evaluate the C:N mass ratios obtained in the results, with higher ratios suggesting a higher amount of chitin (Cherel et al., 2009a). It may be possible to define a C:N ratio beyond which SIA results should be discarded, or to calculate a correction factor based on previous studies [e.g., Post et al. (2007)]. Results are usually considered unusable if C:N ratio values are above 4.0 but it may be appropriate to use results from part of the beak (i.e., the more heavily pigmented part) or to relax the rule if the study is on a rare species. This is particularly important when analysing beaks of juvenile cephalopods for which much of the structure is transparent, thus with higher chitin concentration than in the fully tanned beaks of adults (Clarke, 1986; Cherel et al., 2009a). This limitation can be overcome by removing the transparent part of the beak (Matias et al., 2019; Staudinger et al., 2019) or by using CSIA-AA which is not dependent on the amount of chitin in the beaks (Cherel et al., 2019; Woods et al., 2022). Regarding the use of different sections of the beaks for SIA, it should be noted that values obtained from the tip of the rostrum, sometimes considered as a proxy for juvenile life, result from a mixture of beak material deposited in early life stages and new beak material deposited over the life of the individual (Queirós et al., 2018). This suggests that in species that migrate and increase their trophic position throughout their life, *δ* ^13^C values are higher or lower depending on the habitat occupied by the adult, and *δ* ^15^N values are higher due to the higher trophic position in the later life-stage (Queirós et al., 2018).

### Trace elements

Trace elements occur naturally in the environment, yet their concentrations are increasing due to anthropogenic activities (Sen and Peucker-Ehrenbrink, 2012). These elements can be essential (e.g., copper, iron, zinc) or non-essential (e.g., cadmium, mercury, lead), with both being potentially toxic at a given concentration (Jakimska et al., 2011). They may bio-accumulate throughout the life of individuals and some of them can bio-magnify through the food webs, with the main uptake being by prey ingestion (Szynkowska et al., 2018). Because of their importance in the food web, trace element concentrations have been extensively studied in cephalopods (Bustamante et al., 2000; Seixas et al., 2005; Pierce et al., 2008; Lischka et al., 2020; Seco et al., 2020). However, they have only recently been measured in cephalopod beaks (Fang et al., 2019; Lin et al., 2019; Northern et al., 2019). While numerous studies measured the concentration of mercury in beaks (Xavier et al., 2016; Matias et al., 2019; Queirós et al., 2020a), studies analyzing the concentration of other trace elements are rarer. Northern et al. (2019) measured the trace element concentrations in three different sections of *Moroteuthopsis ingens* lower beaks using both solution based inductively coupled plasma mass spectrometry (SB-ICP-MS) and laser ablation inductively coupled plasma mass spectrometry (LA-ICP-MS) methodologies. Both techniques were able to measure the concentrations of at least 23 elements, though LA-ICP-MS was able to detect three additional elements (i.e., Be, Y and Zr), that SB-ICP-MS did not detect, and also found more variability between beak sections (Northern et al., 2019). More studies are needed to understand whether this inequality is related to the methodology or to differences between the studied specimens.

For trace elements analyses, in contrast to SIA, the tip of the rostrum cannot be considered a proxy for the juvenile life phase as results obtained in this section are similar to those obtained in the end of the hood (Queirós et al., 2020a). Here, the anterior section of the hood is the most appropriate to study this life phase (Queirós et al., 2020a). Analyses of trace elements on beaks are useful for: 1) ecotoxicological studies (Xavier et al., 2016; Queirós et al., 2020a), 2) biogeographical studies to determine the distribution and migration of individuals (Fang et al., 2019; Northern et al., 2019), 3) ecological studies to evaluate the individuals trophic ecology and diet changes during ontogeny (Matias et al., 2019), and 4) the identification of cryptic species (Fang et al., 2021c). As cephalopods are usually widely distributed all around the world, measuring the concentrations of the different trace elements in beaks can also be used for biomonitoring the variability of contamination across ocean basins and, by using beaks preserved in collections, it may allow an evaluation of how concentrations of these elements have changed over time. Moreover, as performed in other taxa, such as bivalves and fish (Campana et al., 1994; Gomes et al., 2016), the analysis of trace elements over the life of individuals in beaks can be used to study the connectivity between different areas to help in the conservation of species and management of cephalopod fisheries.

It is worth noting that, when analysing trace element concentrations in cephalopod beaks, the resulting concentrations are significantly lower than those in other tissues such as muscle, digestive gland, or gills (Xavier et al., 2016; Matias et al., 2019; Matias et al., 2020). Although a previous study found a relationship between mercury concentrations in beaks and muscle in one Antarctic octopod species, which would suggest the former as a potential proxy for the mercury concentration of the flesh, no such relationship was found in other species (Matias et al., 2020). However, the analyses of trace element concentrations in different sections of the beaks may allow to determine differences (i.e., ratios) of elements concentrations in different life stages (Queirós et al., 2020a). For example, mercury concentrations on beaks suggest that adults of *M. longimana* have twice as more mercury than juveniles, which also may happen in the muscle (Queirós et al., 2020a). Ultimately, element concentrations measured in beaks can be used to estimate the concentration of the element that might be transferred from the prey to the predator.

Measuring trace element concentrations in cephalopod beaks does have some limitations. As beaks tend to have lower concentrations of the target elements, the detection limit of the techniques can be a problem, especially when looking for minor elements and when analysing subsections of the beaks for trace metal analyses. This should not be a problem when analysing entire beaks (except if they are from small species or very young individuals). When a specific equipment is available for an element, e.g., Advanced mercury analyser to mercury, its use should be prioritised as it is more sensitive to the element and enables the measurement of lower concentrations. Otherwise, LA-ICP-MS is probably the best option as it has a lower detection limit than other techniques (Fang et al., 2019; Northern et al., 2019; Fang et al., 2021c), and is thus suitable to determine concentrations of minor elements or to measure the concentrations of trace elements in small sections of the beaks. The amount of sample needed to perform some trace element analyses can also be a problem for both entire small beaks and/or beak sections. Here, as for SIA, pooling beaks with similar size, origin and time of sampling is a possible solution (Queirós et al., 2020a; Guímaro et al., 2021). These limitations also apply to the emerging compound-specific trace element analyses (Weiss et al., 2008; Wiederhold, 2015). Emerging compound-specific trace element analyses, such as mercury stable isotopes, can offer a great opportunity to delineate the vertical habitat of cephalopods, as already shown in sharks (Le Croizier G. et al., 2020; Le Croizier G.l. et al., 2020; Besnard et al., 2021) and seabirds (Renedo et al., 2018). Indeed, when using trace elements to study individual migrations, it is important to know if there is any ontogenetic change in the diet or trophic position, because as trace elements are mostly taken up by diet (Szynkowska et al., 2018), we need to be sure to be sure that changes in trace element concentrations are in fact related to changes of habitat and not with the trophic position of cephalopods.

Beak composition should always be considered in any of the analyses mentioned here. As some trace elements have a higher affinity for proteins, e.g., mercury (Bustamante et al., 2006), the changing protein:chitin ratio along the beak can potentially influence the result. Hence, it is important to be careful when analysing the results, especially when comparing different beak sections or beaks from different species for trace elements, sizes and maturation states (Queirós et al., 2020a). As with SIA, this limitation can be overcome with the compound specific trace elements analysis (Renedo et al., 2018; Cherel et al., 2019; Whiteman et al., 2019; Besnard et al., 2021).

### DNA analyses

Genetic analysis plays an important role in the study of cephalopod systematics and evolution (Boyle and Rodhouse, 2005; Strugnell et al., 2011; Allcock et al., 2015; Bolstad et al., 2018). It has also been used to identify cephalopod flesh found in the stomachs of predators as well to study the role of cephalopods as predators (Deagle et al., 2005; Braley et al., 2010; Hoving et al., 2014; Olmos-Pérez et al., 2017; Fernández-Álvarez et al., 2018; Queirós et al., 2021b). These previous studies used the flesh, both muscle and buccal mass, of individuals from collections, or that were captured or washed up on the shore (Bolstad et al., 2018; Queirós et al., 2020b). As some cephalopods, in particular oceanic squids, tend to easily avoid capture and many predators prey/scavenge on them, extracting DNA from the beaks would be an important tool as it would facilitate the identification of some species whose beaks are very similar [e.g., *Histioteuthis eltaninae* and *H. atlantica* (Xavier and Cherel, 2021)], the identification of beaks belonging to undescribed species [e.g., Oegopsida sp. A, *Taonius* sp. (Clarke), *Onychoteuthis* sp. B (Imber) (Cherel et al., 2004; Cherel et al., 2011; Cherel, 2020)], and to study the phylogeny and taxonomy of these hard-to-catch species.

As far as we know, only one study was able to extract DNA from cephalopod beaks (Vecchione et al., 2009). These authors extracted DNA from a beak of *Muusoctopus thielei* collected from a whole specimen. Another successful tentative trial was made in the beaks of *Architeuthis dux* but the amount of extracted DNA was not enough to follow up with further research at the time (Tom Gilbert, personal communication). In contrast, Xavier et al. (2016) did not succeed in extracting DNA from beaks of *M. longimana* and *Filippovia knipovitchi*. When comparing the studies of both Vecchione et al. (2009) and Xavier et al. (2016) to understand what could influence DNA extraction, one primary difference is that the former study used fresh beaks retrieved from the individual, while the latter used non-fresh beaks from predators’ diet. After spending time in the stomach, the beaks lost their transparent part which is the area closer to the beccublast cells that synthesise the beak proteins (Tan et al., 2015). Further differences can also be found in the methodology used in both studies (Vecchione et al., 2009; Xavier et al., 2016). We believe that it is worthwhile to continue to attempt the extraction of DNA from cephalopod beaks, although we suggest that studies should focus on the transparent parts of the beaks, rather than the entire, fully sclerotized, beak. Nevertheless, the use of the transparent part of the beak could be a limitation for these methodologies in smaller species in which the transparent part is reduced, as well as in beaks from predators’ stomachs since the transparent part disappears with the action of the gastric acids (Clarke, 1986; Duffy and Jackson, 1986). Furthermore, we suggest trying different methodologies, including approaches that have been used to successfully extracted DNA from other difficult-to-handle issues e.g., bones or fossils (Vecchione et al., 2009; Xavier et al., 2016; Campos and Gilbert, 2019; Modi et al., 2021).

### Structural analysis

The rostrum of cephalopod beaks is among the hardest and stiffest fully organic materials on Earth, while the lateral walls and wing areas of these beaks are generally soft and flexible (Miserez et al., 2008). Indeed, the beak rostrum can be harder than engineering polymers and present an intermediate response to blunt abrasion (Miserez et al., 2007). Because of these characteristics, beaks are seen as an inspiration for new engineering protein-based and environmentally load-bearing polymers (i.e., polymers that can support great amounts of weight) that can replicate properties of living organisms (Miserez et al., 2010; Linder, 2015; Sun et al., 2020). Furthermore, there is also an interest in chitosan, a biopolymer obtained from chitin (Miserez et al., 2008), which has applications in the food industry, pharmaceuticals, textiles and biotechnology (Morin-Crini et al., 2019). However, for such compounds to be replicated, it is important to understand how they are formed and what gives the beaks their unique properties since they do not contain metal ions, minerals, or halogens, which typically confer hardness and stiffness to biomaterials [e.g., Zinc (Zn) ions on polychaete jaws (Miserez et al., 2007; Linder, 2015)]. The determination of beak characteristics can also be used in the study the trophic ecology (by providing insights into what kind of prey could be eaten) (Matias et al., 2019).

Several studies analysed the structure and mechanical properties of cephalopod beaks, most of them using those of the jumbo squid *Dosidicus gigas* (Miserez et al., 2007; Miserez et al., 2008; Miserez et al., 2010; Tan et al., 2015)*.* To our knowledge, apart from these studies, only Matias et al. (2019) have studied the mechanical properties of cephalopod beaks, using two Antarctic octopod species, *Pareledone turqueti* and *Adelieledone polymorpha*. The methodology used by all these studies was very similar [i.e., optical and scanning electron microscopy and high-resolution microcomputed tomography to determine the microstructure and structural features, x-ray diffraction to study the density, nanoindentation test to determine the beak mechanical properties and single-edge notched tension (SENT) to determine the fracture toughness]. In contrast to previous methodologies, only one limitation was found, and it was related to the size of the beak and its suitability for some tests (e.g., SENT test). This is why the authors decided to use very large *D. gigas* beaks (Miserez et al., 2007). The similarity between the techniques used to date suggests that future studies could use the same approach to study the structural properties of beaks, facilitating comparisons, providing insights into the ecology of the species and inspiring the engineering of new materials. Indeed, different results obtained for three species showed different hardness in the following sequence, from hardest to least hard: *D. gigas* > *P. turqueti* > *A. polymorpha* (Miserez et al., 2007; Matias et al., 2019).

### Proteomics

The study of an organisms’ proteome can assume a major role in understanding how species will react to climate change, pollutants and other environmental stressors (Nunn and Timperman, 2007; Braconi et al., 2011; Tomanek, 2014). Indeed, previous studies showed that temperature, toxic trace elements, food limitation, or hormones can all affect the proteins in molluscs, as well as their amino acid pool (Veldhoen et al., 2012; Clark et al., 2017). Furthermore, an organisms’ proteome can help in species’ identification (Mazzeo and Siciliano, 2016). A proteomic approach has been used in cephalopods to understand their colours, toxins, host-parasite relationships and their immune system (Gestal and Castellanos-Martínez, 2015; Roumbedakis et al., 2018; Albertin and Simakov, 2020; Gonçalves and Costa, 2021). These studies used tissues such as skin (Crookes et al., 2004), slime (Caruana et al., 2016), saliva (Cornet et al., 2014), and in cuttlefish cuttlebones (Pabic et al., 2017).

In a pioneering study, Miserez et al. (2008) carried out the first analysis of the proteins in cephalopod beaks. Using beaks of the jumbo squid *D. gigas,* they found differences in the amino acids composition between the tanned and untanned areas (Miserez et al., 2008) and showed that the stiffness of the beaks was linked to the amount of certain proteins, identifying l-3,4-dihydroxyphenylalanine-histidine (dopa-His) as providing mechanical strength to the beak material. The authors subsequently found many cross-links were actually based on (His)-4-methylcatechol and not dopa-His (Miserez et al., 2008; Miserez et al., 2010). Following these studies, Tan et al. (2015) combined different transcriptomic (using mRNA from beccublast) and proteomic techniques to study the proteins in cephalopod beaks. They found the presence of two major families of proteins: the chitin-binding proteins (DgCBPs) and the histidine-rich beak proteins (DgHBPs), the former new to science (Tan et al., 2015). However, the precise distribution of each protein in the beak remains unknown, and only estimations using its wet mass are available (Tan et al., 2015). Despite this major step, it is still true that very little is known about the proteome of cephalopod beaks, especially because these studies focused only on one species and, as proteins are related to stiffness and beaks of different species have different stiffness structural analyses (Miserez et al., 2008), it is important to know if these proteins are the same for all species. Additionally, the presence of stress marks during the formation of beaks suggests changes during their formation that can be related to proteins (Perales-Raya et al., 2014a).

The study of cephalopod beak proteins is important to protein engineering as they can be a model for liquid-liquid phase separation (Sun et al., 2020). Another advantage of studying beak proteins, as with other techniques, is their availability in collections and the possibility of using predators as biological samplers. This suggests that cephalopod beaks can be used to study environmental changes through time but also be useful to study impacts of environmental stressors in species that are challenging to sample. As proteomics can also be used in evolutionary and ecological studies (Diz et al., 2012), it also has the potential to help in the study of cephalopod evolution. However, there are known limitations to studying beak proteins, and major uncertainties since this is a very new field of study. The major limitation found by Tan et al. (2015) is that classic protein extraction protocols do not work on beaks. They overcame this limitation by using non-enzymatic reagents that cleave peptide bonds, releasing them from the beak structure (Tan et al., 2015). However, other strategies are needed to extract beak proteins without destroying them. Furthermore, it is still unknown whether different methods of preservation have different effects on proteins and whether proteins are still present in beaks from museum collections.

## Future challenges in research in cephalopods beaks

Regarding the use of beaks for taxonomy, some relevant key features, limitations and perspectives are outlined below. Most previous investigations used lower beaks only (Clarke, 1986; Xavier et al., 2007a; Xavier et al., 2015). However, discarding upper beaks is a potential loss of information that was highlighted in subsequent investigations (Cherel et al., 2000; Cherel et al., 2017). Hence, both lower and uppers beaks should be included in future studies (Xavier et al., 2011). Also, care is needed when using names of species assigned to beaks in older publications, due to a combination of past misidentifications and subsequent improvements in both beak identification and cephalopod taxonomy over the last few decades (Cherel, 2020, 2021). Also, beaks are routinely used in the studies of trophic ecology of predatory species to estimate prey length and mass. However, regression formulas of relationships between beak size and length and mass of cephalopods are lacking for the majority of species. Extensive collection and routine publishing of such data is an essential task in cephalopod research in the future. Another challenge is that, despite the recent global revision of some families (e.g., Onychoteuthidae) (Bolstad, 2010; Bolstad et al., 2018), cephalopod taxonomy is still problematic: the chaotic state of some taxa precludes identifying beaks to the species level with confidence in many cases (e.g., Brachioteuthidae, Chiroteuthidae). Improvement in beak identification requires that both taxonomic revision and the description of new species include drawings and/or photos of the lower and upper beaks, ideally from early stages to mature adults. It also requires exploring new methods to extract DNA from biological samples in poor condition. In most cases, as reported above, conventional procedures fail to extract DNA from partly digested cephalopod flesh, thus preventing the use of buccal masses from food samples to confirm/inform and thus improve cephalopod identification based on the corresponding beaks. This loss of information is unfortunate because most oceanic squids are notably difficult to catch using traditional means, while some species form a significant part of predators’ diet. In a few cases, the reverse is true, with beaks helping to solve systematic issues. For example, conventional examination of squid morphology and anatomy failed to find differences between *Histioteuthis bonnellii bonnellii* and *H. b. corpuscula* (Voss et al., 1998) [presently considered synonym: *H. bonnellii* (MolluscaBase, 2022)], while both beak morphology and size clearly indicate that they belong to different taxa (Clarke, 1980), a finding that merits further genetic investigation using the new generation of efficient DNA tools. Finally, identifying beaks from their morphology is time-consuming and needs expertise. We thus recommend getting expert advice before attributing a species name to a beak (Xavier and Cherel, 2021). Unfortunately, the most important bottleneck of the method now is the low and decreasing number of experts, meaning that efforts must be made to train early career researchers to identify cephalopod beaks from their morphology. The value of conventional photographic guides of cephalopods beaks for training and species identification should be recognized. Existing beak identification guides have covered only minor part of cephalopods diversity and their regional morphological variability. Therefore, the development of new extended guides or web-based solutions (see examples above) are still essential. Additionally, simultaneous collaborative efforts focused on collection of beaks, their photographs and DNA samples deposited as public web-based resource may also serve as important reference database for cephalopod identification in the future, as it already occurs with fish (i.e., AFORO as an example for collaborative otoliths database (http://aforo.cmima.csic.es/) (Lombarte et al., 2006).

A promising alternative or supplement to identification of cephalopods based on the morphology of their beaks is the development of a software for geometric morphometric-based automatic identification of the beaks. However, this approach also has some limitations. The first issue to solve is the development of efficient methods of acquisition of images for processing. At the moment, there is no agreement about the equipment to use, from which angles to record images, and how to take photos of the beaks. Some authors used 2-D images of the beak’s lateral view (Fang et al., 2017; Jin et al., 2017; Tan et al., 2021), others used a complex system of mirrors to obtain combined 2-D images of frontal and lateral views (Crespi-Abril et al., 2010), and others used a combination of underwater photogrammetry and CT scanning to obtain 3-D images of beaks (Roscian et al., 2022). As results of studies based on 2-D images suggest, this is enough for routine identification of abundant species (Tan et al., 2021), although in-depth ecological, paleontological, or taxonomic studies may demand more complex method (Roscian et al., 2022). We argue that the use of beaks for identification purposes demands a simple approach, which can be adopted both in the field and in the laboratory. From this point of view, acquisition of 2-D images of the beak’s lateral view seems more promising (Fang et al., 2017; Tan et al., 2021), but further studies on the loss of information and accuracy, when in using images of only the lateral view, are still needed.

Another issue is the selection of methods for analysis of images. Landmark-based methods of analysis have plenty of advantages in this regard, specifically when it comes to preserving ecologically or taxonomically meaningful information, but approach requires experienced users. On another hand, outline-based methods more suitable for automation of the process, since they do not require the user to select the points of interest or otherwise fine-tune the analysis. Thus, the software for automatic identification of the cephalopods based on the shape of their beaks may be based on Fourier or wavelet transformation, similar to the system developed for fish identification (Lombarte et al., 2006). It should be noted that accuracy of such an approach to identification may be improved if supplemented by the analysis of beak’s pigmentation (Fang et al., 2017), or if only the pigmented part is analysed (Lishchenko and Jones, 2021).

Further studies on a large number of taxa, testing the phylogenetic signal carried by beak shape will be necessary to establish the level of accuracy that could be achieved in identification. Moreover, as 3-D reconstruction is time-consuming and more complex than 2-D analyses, recent work using 3-D geometric morphometrics, which allows the complexity of beak shape to be better captured, has shown that the phylogenetic signal of upper and lower beaks is significant but moderate and that it cannot explain all the morphological variation in beaks on its own (Roscian et al., 2022). On the other hand, cephalopod beaks remain complex to image in three dimensions without damaging them. The use and improvement of advanced imaging technologies such as X-ray tomography and underwater photogrammetry on small objects will allow most species to be digitized (Roscian et al., 2021; Ziegler and Sagorny, 2021; Roscian et al., 2022). Nevertheless, the digitization of specimens is a long task and the accumulation and accessibility of the data to the community is a major challenge in achieving a comprehensive sampling of cephalopod 3-D model beaks. These 3-D models could indeed allow us to thoroughly renew our understanding of the signal carried by the shape of the beaks and be very complementary to 2-D analyses. Recently, Roscian et al. (2022) showed, using 3-D geometric morphometrics, that there is a likely link between ecological parameters such as habitat and trophic level and beak shape. These outcomes are in accordance with those obtained for squid and octopod paralarvae indicating a relationship between beak shape variation and diet shifts (Franco-Santos et al., 2014; Franco-Santos and Vidal, 2014; Franco-Santos and Vidal, 2020).

The study of developmental features of the beak during the early ontogeny of cephalopods is a nearly unexplored field of study. Much remains to be investigated in relation to the developmental pattern, shape and chemical composition of the beak and its relationship with the feeding ecology during early life. These studies encourage further research into the analysis of beak shape in relation to the ecology of the taxa, to test whether adaptive traits can be identified in these structures at any phase of the life cycle. To achieve this goal, it will be necessary not only to have many 3-D models but also to collect more data on the diets, habitats and trophic positions of species for which information remains scarce or poorly known, such as some deep-sea octopods or squids (i.e., some members of the genus *Opisthoteuthis*, the pelagic *Vitreledonella richardi* or *Asperoteuthis lui*). This approach will open new opportunities to study the evolutionary history of cephalopods, as it will be possible to integrate fossil forms. Although the contribution of fossil beaks to the reconstruction of the evolutionary history of coleoids is challenging due to the deformations of the fossil record and their rarity, methods are now available to overcome these problems (Hughes and Jell, 1992; Schlager et al., 2018; Demuth et al., 2022). Quantitative comparative analyses of modern and fossil beaks are now achievable and should produce significant advances in paleoecological interpretation of selected fossil forms.

Regarding using beaks in age and growth studies, some key relevant features, limitations and perspectives of the field are outlined below. When working on age determination of a species for the first time, exploration of both lateral wall surfaces and rostrum sagittal sections is mandatory. Increments do not always present regular pattern in the lateral wall surfaces, but they usually do so in the rostrum sagittal sections. Moreover, both upper and lower beaks need also to be examined to select the most suitable for the species of interest. Some species exhibit high erosion and irregular increment sequences in lateral wall surfaces of upper beaks (e.g., *Architeuthis* and *Moroteuthopsis*), whereas in others (e.g., *Octopus vulgaris*) the upper beaks are the most suitable for age estimation. When using beaks from cephalopod predators (stomach contents), the loss of material from the external border of these beaks might be substantial, presenting problems if the lateral wall surfaces are used for age estimation, therefore the information on how long it takes for the transparent part of lateral walls to disappear would help to prevent age underestimations. On the other hand, it is necessary to know how long the beak has been in the stomach to have a proxy of the death day of the specimen. These issues are covered in depth further down since further research is desirable. Age validation experiments are scarce in beaks, but they are required to confirm the periodicity of deposition of the growth increments in the species of interest. Mark-recapture methods in wild populations are expensive but others such as captive experiments, using marking or known-age specimens, are suitable for age validation when aquaculture facilities are accessible and the species can live in captivity for some time. It is also important to validate the age of the first increment, not only the temporal deposition of increments, to obtain reliable age estimations. When the species of interest is unsuitable for validation experiments (e.g., deep-water species), cross-verification by comparing with other validated structures (e.g., statoliths in squids or vestigial shells in octopuses), is an alternative.

The observation, counting and analysis of daily increments is time-consuming. The life-mode approach to performing semi-automated counts is advisable but not usually available in current image analysis systems. Artificial intelligence could provide a useful tool to save time and improve the detection and count of increments since it could “learn” from the images previously analysed by experienced readers. Finally, the cumulative width of the daily increments could be a potential tool to estimate growth in the wild, before capture (e.g., Perales-Raya et al. (2020) for *Architeuthis dux*; Queirós, Bartolomé, Xavier and Perales-Raya, unpublished for *M. longimana*). Preliminary results on reared *O. vulgaris* used correlations between cumulative widths and body mass to estimate the growth in the wild, before capture (Perales-Raya, Bartolomé, Márquez, Felipe and Almansa, unpublished data). Moreover, future research should also further evaluate beak growth under warming (e.g., climate change scenarios) under laboratory conditions, which is known to cause thermal stress in cephalopod beaks (E.g., in *Octopus vulgaris*) (Perales-Raya et al., 2014a) (Perales-Raya et al., 2014b), in order to validate the magnitude of such beak marks and their ecological implications.

The application of different chemical and structural analyses on cephalopod beaks to study different aspects of the species and individual life-cycle is increasing, though with some techniques being well-established in cephalopods (e.g., stable isotopic analyses), while others are still in development (e.g., proteomics). Nevertheless and independently of whether a method is “established,” all methods present issues and challenges that should be addressed in the future. Stable isotopic analysis, as is the case for most of the techniques applied on beaks, is dependent on the chitin:protein ratio, which varies throughout the beak. Future studies should evaluate whether the variation of the chitin:protein ratio is similar across species. Furthermore, as a higher proportion of chitin results in lower *δ* ^15^N values, a correction factor for chitin, similar to those used in stable isotopic analyses on muscle for lipids (Hobson and Cherel, 2006), should be found to enable the direct comparison of *δ* ^15^N values between different sections of the beak and different life-stages and to compare with other tissues. Several studies applied SIA on beaks from predators’ stomachs, with most of these determining the habitat using known gradients of *δ* ^13^C values in the environment (Cherel and Hobson, 2005; Guerreiro et al., 2015; Abreu et al., 2020), especially when using different sections of the beak (Guerra et al., 2010; Queirós et al., 2018). If the capture location is known, the *δ* ^13^C value of the last formed beak material could be used as a baseline that helps to determine the movement of the individual. However, to have an idea of the capture location if the sample came from stomach contents, it is important to know how long the beak remained in the stomach. Although there is some information on the amount of time a beak can stay in a predator stomach (Ashmole and Ashmole, 1967; Clarke, 1980; Jackson and Ryan, 1986; Gales and Cheal, 1992; Xavier et al., 2011), future experimental studies are needed to understand the average time a beak takes to be egested in relation to the type of predator and how long it takes for transparent parts of a beak to disappear, depending on both its own size and darkening stage as well as on the predator biology. Consequently, it is essential for diet studies to distinguish the beaks that are from recently eaten prey (i.e., beaks recently consumed by predators that still have flesh attached, beaks in buccal masses or from complete or partially digested specimens) from those which have been in the stomach a long time and may be eroded (i.e., beaks without transparent parts or flesh attached).

SIA is also dependent on a baseline value and, despite some previous studies determining POM isotopic values for the different areas or modelling these values, future studies should update these values, partly because they may change over time, as well as using beaks from individuals with known capture location to create an isoscape specifically for cephalopod beaks. Apart from the baselines, future studies should focus on establishing a specific trophic enrichment factor for cephalopod beaks (ideally for each cephalopod species in a given region) that enables the study of the trophic level of an individual using an enrichment factor adapted for these structures rather than general enrichment factors that are common for all marine organisms.

Regarding the trace elements analyses, further studies should investigate in detail whether the differences found between results from different techniques used in previous studies (i.e., SB-ICP-MS and LA-ICP-MS) (Northern et al., 2019), are a consequence of the technique or simply a function of which beaks were used. It is also important that future studies investigate the relationship between element concentrations in the beaks and those found in other tissues of the individual. Due to the different chitin:protein ratio along the beak and the different affinity that some elements have with proteins, it is important that future studies evaluate how the change in beak composition can affect the concentrations of the different elements, and look in detail at the rostrum as this part of the beak may be prone to accumulate high levels of some elements, not working as a proxy for the juvenile life-phase as it does in SIA (Queirós et al., 2020a). Additionally, it is important to evaluate the usefulness of beaks as proxy of environmental pollution, such as studying these trace elements in beaks related to pollution (e.g., increased mercury levels due to anthropogenic sources), to provide a proxy of pollution in cephalopods.

 Concerning the most recent techniques applied to beak structure and protein composition, we suggest that the various available methodologies are tested in order to find which are the most efficient. Considering previous studies, we suggest that future research aiming to extract DNA from (fresh) beaks from predators’ stomachs that still have their transparent parts, as well as the use of techniques that have been shown to recover DNA material from fossils or bones. Regarding structure analyses, we suggest that future studies should aim to develop an understanding of the interspecific variability in the beak structure and how it can influence, for example, the diet of each species. The study of the beak proteins is, as far as we know, one of the most recent techniques that has been applied to these structures. Based on its importance for beak composition and its influence on the ecology of the species, we suggest that new techniques should be explored: techniques used in the past able to retrieve proteins, though destroying the beaks, should be applied in other species to understand the inter-species variability. Future studies should also explore the effect of the different preservation methods on the beak proteins to evaluate whether beaks in museum collections can be used in these studies.
