# Corrigendum: The significance of cephalopod beaks as a research tool: An update

**DOI:** 10.3389/fphys.2023.1140110

**Published:** 2023-01-25

**Authors:** José C. Xavier, Alexey V. Golikov, José P. Queirós, Catalina Perales-Raya, Rigoberto Rosas-Luis, José Abreu, Giambattista Bello, Paco Bustamante, Juan C. Capaz, Valerie H. Dimkovikj, Ángel F. González, Hugo Guímaro, Airam Guerra-Marrero, José N. Gomes-Pereira, Jorge Hernández-Urcera, Tsunemi Kubodera, Vladimir Laptikhovsky, Evgenia Lefkaditou, Fedor Lishchenko, Amanda Luna, Bilin Liu, Graham J. Pierce, Vasco Pissarra, Elodie Reveillac, Evgeny V. Romanov, Rui Rosa, Marjorie Roscian, Lisa Rose-Mann, Isabelle Rouget, Pilar Sánchez, Antoni Sánchez-Márquez, Sónia Seixas, Louise Souquet, Jaquelino Varela, Erica A. G. Vidal, Yves Cherel

**Affiliations:** ^1^ Department of Life Sciences, Marine and Environmental Sciences Centre/ ARNET–Aquatic Research Network, University of Coimbra, Coimbra, Portugal; ^2^ British Antarctic Survey, Natural Environment Research Council, Cambridge, United Kingdom; ^3^ GEOMAR Helmholtz Centre for Ocean Research Kiel, Kiel, Germany; ^4^ Instituto Español de Oceanografía (IEO,CSIC), Santa Cruz de Tenerife, Spain; ^5^ CONACYT -Tecnológico Nacional de México/I. T., Chetumal, Quintana Roo, Mexico; ^6^ Mola di Bari, Italy; ^7^ Littoral Environnement et Sociétés (LIENSs), UMR 7266 CNRS-La Rochelle Université, La Rochelle, France; ^8^ Institut Universitaire de France (IUF), Paris, France; ^9^ Center of Marine Sciences, University of Algarve, Campus de Gambelas, Faro, Portugal; ^10^ Department of Marine Science, Coastal Carolina University, Conway, SC, United States; ^11^ Instituto de Investigaciones Marinas (CSIC), Vigo, Spain; ^12^ IU-ECOAQUA, University of Las Palmas de Gran Canaria, Edf. Ciencias Básicas, Campus de Tafira, Las Palmas de Gran Canaria, Spain; ^13^ Atlantic Naturalist Association, Horta, Portugal; ^14^ National Museum of Nature and Science, Tokyo, Japan; ^15^ Centre for Environment, Fisheries and Aquaculture Science (CEFAS), Lowestoft, United Kingdom; ^16^ HCMR, Hellenic Centre for Marine Research, Athens, Greece; ^17^ Laboratory for Ecology and Morphology of Marine Invertebrates, A.N. Severtsov Institute of Ecology and Evolution of the Russian Academy of Sciences, Moscow, Russia; ^18^ Department of Ecology and Animal Biology, Faculty of Marine Sciences, University of Vigo, Vigo, Spain; ^19^ College of Marine Sciences, Shanghai Ocean University, Shanghai, China; ^20^ MARE—Marine and Environmental Sciences Centre/ ARNET–Aquatic Research Network, Laboratório Marítimo da Guia, Faculdade de Ciências, Universidade de Lisboa, Cascais, Portugal; ^21^ Centre Technique de Recherche et de Valorisation des Milieux Aquatiques (CITEB), Le Port, Île de la Réunion, France; ^22^ Centre de Recherche en Paléontologie-Paris (CR2P), CNRS, Sorbonne Université, Paris, France; ^23^ University of South Florida, College of Marine Science, St. Petersburg, FL, United States; ^24^ Institut de Ciènces del Mar, CSIC, Psg. Marítim de la Barceloneta, Barcelona, Spain; ^25^ Universidade Aberta, Rua Escola Politécnica, Lisboa, Portugal; ^26^ Department of Mechanical Engineering, Faculty of Engineering Science, University College London, London, United Kingdom; ^27^ Center for Marine Studies—Federal University of Parana (UFPR), Pontal do Paraná, PR, Brazil; ^28^ Centre d’Etudes Biologiques de Chizé, UMR 7372 du CNRS-La Rochelle Université, Villiers-en-bois, France

**Keywords:** Cephalopod ecology, beak taxonomy/composition/morphology/microstructure/ paleontology, cephalopod trophic dynamics, cephalopod population dynamics, cephalopod ecotoxicology

In the published article, there was an error in the author list, and author Jorge Hernández-Urcera was erroneously excluded. The corrected author list appears below.

“José C. Xavier^1,2,^*^,^ Alexey V. Golikov^3^, José P. Queirós^1,2^, Catalina Perales-Raya^4^, Rigoberto Rosas-Luis^5^, José Abreu^1,2^, Giambattista Bello^6^, Paco Bustamante^7,8^, Juan C. Capaz^9^, Valerie H. Dimkovikj^10^, Angel F. González^11^, Hugo Guímaro^1,2^, Airam Guerra-Marrero^12^, José N. Gomes-Pereira^13^, Jorge Hernández-Urcera^11^, Tsunemi Kubodera^14^, Vladimir Laptikhovsky^15^, Evgenia Lefkaditou^16^, Fedor Lishchenko^17^, Amanda Luna^18^, Bilin Liu^19^, Graham J. Pierce^11^, Vasco Pissarra^20^, Elodie Reveillac^7^, Evgeny V. Romanov^21^, Rui Rosa^20^, Marjorie Roscian^22^, Lisa Rose-Mann^23^, Isabelle Rouget^22^, Pilar Sánchez^24^, Antoni Sánchez-Márquez^24^, Sónia Seixas^1,25^, Louise Souquet^26^, Jaquelino Varela^20^, Erica A. G. Vidal^27^ and Yves Cherel^28^”

In the published article, there was an error in affiliation 28. Instead of “Centre d’Etudes Biologiques de Chizé, UMR 7372 du CNRS-La Rochelle Université, La Rochelle, France”, it should be “Centre d’Etudes Biologiques de Chizé, UMR 7372 du CNRS-La Rochelle Université, Villiers-en-Bois, France.”

The authors apologize for these errors and state that this does not change the scientific conclusions of the article in any way. The original article has been updated.

